# Mixed functional microarchitectures for orientation selectivity in the mouse primary visual cortex

**DOI:** 10.1038/ncomms13210

**Published:** 2016-10-21

**Authors:** Satoru Kondo, Takashi Yoshida, Kenichi Ohki

**Affiliations:** 1 Department of Molecular Physiology, Graduate School of Medical Sciences, Kyushu University, Fukuoka 812-8582, Japan; 2 CREST, Japan Science and Technology Agency, Kawaguchi 332-0012, Japan; 3 Department of Physiology, The University of Tokyo School of Medicine, Tokyo 113-0033, Japan

## Abstract

A minicolumn is the smallest anatomical module in the cortical architecture, but it is still in debate whether it serves as functional units for cortical processing. In the rodent primary visual cortex (V1), neurons with different preferred orientations are mixed horizontally in a salt and pepper manner, but vertical functional organization was not examined. In this study, we found that neurons with similar orientation preference are weakly but significantly clustered vertically in a short length and horizontally in the scale of a minicolumn. Interestingly, the vertical clustering is found only in a part of minicolumns, and others are composed of neurons with a variety of orientation preferences. Thus, the mouse V1 is a mixture of vertical clusters of neurons with various degrees of orientation similarity, which may be the compromise between the brain size and keeping the vertical clusters of similarly tuned neurons at least in a subset of clusters.

## Abstract (web summary)

Primary visual cortical neurons display mostly a salt and pepper arrangement of orientation preferences along the horizontal cortical axis. Here the authors show that a significant subset of minicolumns, one-cell wide arrays of cells arranged along the vertical axis, show similar orientation tuning preferences.

The cerebral cortex is a network of billions of neurons. To understand such a complex network, it is important to understand how the network can be divided into its components. The cortex is partitioned into areas, and each area can be further divided into functional modules^1^. However, the smallest units of functional module organization remain unclear.

Anatomically, two smallest units of cortical architecture have been observed: minicolumns and microcolumns. A minicolumn is a one-cell-wide vertical array of cell bodies running perpendicular to the cortical surface^2^,^3^. In cats and humans, these arrays run through the cortical layers and are regularly distributed, with a spacing of ∼20 μm in between^4^,^5^. The other unit is a microcolumn, which is a group of neurons roughly located vertically, and their apical dendrites (layer 2/3 and 5 pyramidal neurons) make a bundle in the upper layers^6^,^7^. Neighbouring dendritic bundles are separated with a spacing of ∼30 μm in the visual cortex of rats^6^ and of cats^8^. Because some apical dendrites take lateral shifts as these dendrites ascend, minicolumns and microcolumns are not identical^6^. Although it has been repeatedly suggested that minicolumns or microcolumns may be the smallest anatomical module in the cortical architecture^1^,^9^, the functional properties of neurons within minicolumns or within microcolumns have not been investigated, and whether minicolumns serve as functional units for cortical processing remains in debate^1^,^4^,^9^.

In the primary visual cortex (V1) of rodents, single-electrode penetrations did not suggest the vertical organization of neurons with the same orientation preference^10^,^11^,^12^. However, it would be difficult to detect fine structures, such as minicolumns, with the low sampling density of extracellular recording, even if these structures existed. *In vivo* two-photon calcium imaging enabled the study of the spatiotemporal activity pattern of all neurons in a local volume with cellular resolution^13^,^14^. Previous studies with two-photon calcium imaging showed that neurons with different preferred orientations are mixed in a salt and pepper manner parallel to the cortical surface in the primary visual cortex of rodents^14^,^15^,^16^. However, these studies did not reveal whether neurons with similar orientation selectivity exhibit a completely disorganized structure or a vertically organized structure when analyzed three-dimensionally. The analysis of long and narrow cylinder-like minicolumns, whose radius is only 5–10 μm^5^, requires determining the vertical axis strictly, and even small errors may significantly affect the conclusion. Therefore, a decisive conclusion concerning the fine-scale three-dimensional (3D) functional microarchitecture has not been obtained.

In the present study, we examined whether cells with similar response selectivity are arranged as minicolumns or as microcolumns. Recently, an advanced high-speed 3D volume imaging technique enabled the acquisition of data regarding the activity of more than 1,000 neurons in a 3D volume at a time. We used this imaging technique to investigate the 3D functional architecture of neurons in the primary visual cortex of mice with complete sampling of neurons in local volumes, and analysed the similarity of the response selectivity of neurons within minicolumns.

Moreover, we investigated whether neurons in a microcolumn share response selectivity by examining the selectivity of apical dendrites of layer 5 neurons consisting of a single dendritic bundle. Because dendritic calcium signals are dominated by back-propagating action potentials from the soma, the response selectivity of dendrites within a dendritic bundle reflects the response selectivity of neurons within a microcolumn.

## Results

### Anatomical structures of minicolumns in the mouse V1

**Figure 1:**
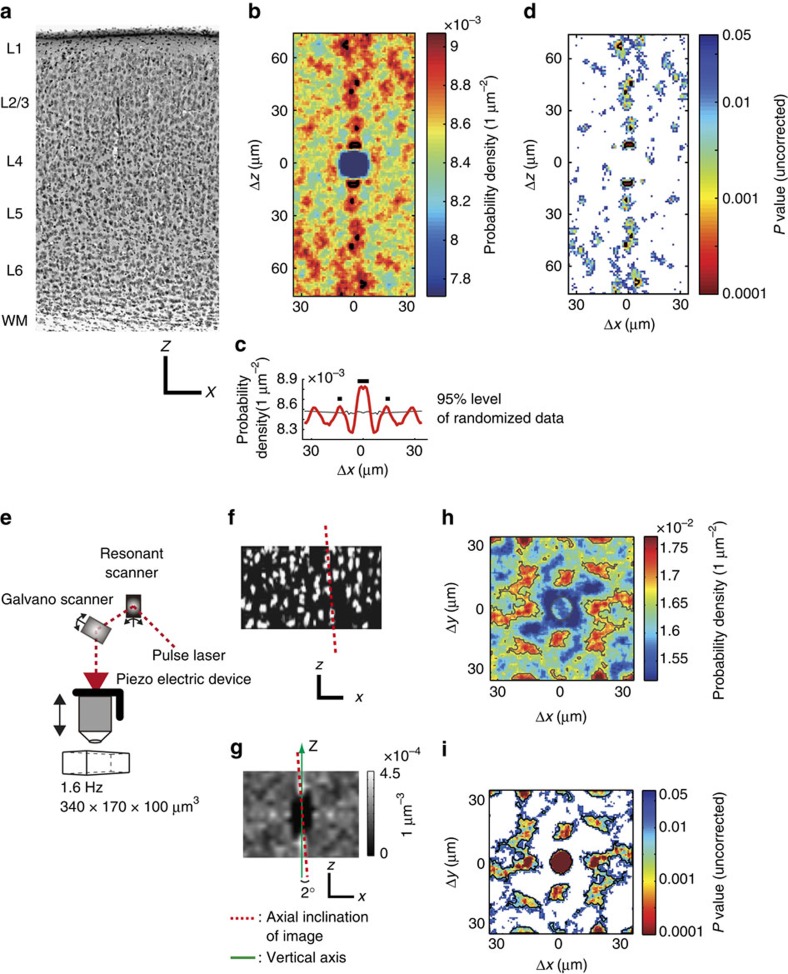
Minicolumnar structure in mouse V1 and three-dimensional imaging. (**a**) Nissl-stained fixed section of the mouse V1. Vertical arrays of cells are visible that span cortical layers. Scale bars, 100 μm. (**b**) A 2D (*x*–z) colour-coded probability density map of neighbouring cell location in Nissl-stained image in **a.** (**c**) 1D projection plot of **b**. The 2D map in **b** is averaged along *z*-axis in the range of ±35 μm. Significant regions (**b**) and peaks (**c**) are indicated by black lines (FDR adjusted *P*<0.05). (**d**) Uncorrected *P* value map corresponding the probability density map (**b**). *P* values are computed by a randomization procedure (see Methods) and indicated by colour code. White area indicates *P*≥0.05. (**e**) A high-speed three-dimensional two-photon microscope used in the present study. The combination of a resonant (*x*-axis) and a galvano scanner (*y*-axis) and a piezo *z*-axis drive enables rapid three-dimensional imaging. (**f**) An *x*–*z* section from a OGB-1-stained volume obtained *in vivo* using two-photon microscopy, showing vertical alignment of cells. The red dotted line shows the axial inclination of the volume. Scale bars, 30 μm. (**g**) A 3D probability density map of neighbouring cells sectioned at *x*–*z* plane. The peaks along the *z*-axis show a vertical alignment of neighbouring cells in the same minicolumns. The angle of inclination of the volume from the *z*-axis (green line) is calculated (red dotted line, 2° in this case) , and the volume image is corrected by tilting. Scale bars, 10 μm. (**h**) A 2D colour-coded probability density map of neighbouring cells in layer 2/3. Several hot spots can be observed, and are regularly spaced at ∼15 μm. The radius of the centre peak is 5.1 μm by fitting with a 2D Gaussian function. Significant areas are contoured by grey lines (FDR adjusted *P*<0.05). (**i**) Uncorrected *P* value map corresponding to the probability density map in **h** . White area indicates *P*≥0.05.

We examined whether anatomical vertical alignment of neurons (minicolumnar structure) exists in the mouse V1, in which it is still controversial. In Nissl staining of a fixed slice section (Fig. 1a), we computed probability density map of neighbouring cell location (Fig. 1b, see Methods). In the probability density map, vertical alignment was evident at least ∼70 μm in length (significant region was indicated by black contour in Fig. 1b. False discovery rate (FDR) adjusted *P* value <0.05. Also see conventional, uncorrected *P* value map in Fig. 1d). We also found that this vertical alignment tended to repeat in the horizontal direction with roughly regular interval (∼15 μm), which was clearly observed in one-dimensional projection plot of the probability density map on the *x* axis (Fig. 1c). Significant peaks are indicated by black lines. FDR adjusted *P*<0.05). These results suggest vertical alignment of cell location and its horizontal periodicity in the Nissl-stained section.

We next examined cell alignment *in vivo* by 3D imaging of two photon microscopy (Fig. 1e). Figure 1f shows *in vivo* images of cell bodies of neurons and of astrocytes in layer 2/3, which were loaded with a calcium indicator, Oregon Green BAPTA-1 acetoxymethyl ester (OGB1-AM). The vertical alignment was also evident but was tilted by a few degrees (red dotted line). The tilt of the alignment was estimated from the 3D probability density map of the locations of all the neighbouring cells (the red dotted line in Fig. 1g) and corrected (see Methods). After correcting the tilt, we constructed two-dimensional (2D) probability density maps of the locations of all the neighbouring cells (Supplementary Fig. 1a,b) and all the neighbouring orientation-selective cells (Fig. 1h and Supplementary Fig. 1c,d) as a function of the horizontal displacement between cell pairs, ignoring vertical displacement (see Methods). The 2D probability density map (Fig. 1h) shows several hot spots spaced at ∼15 μm, indicating that the vertical structures tended to be aligned regularly, with a spacing of ∼15 μm (see Supplementary Fig. 1c,d for the density and number of orientation selective cells around these hot spots). Because the probability density map was calculated between the pairs of cells excepting the pairs of themselves, the values at the centre tended to be lower than the surrounding hot spots. Statistical analysis revealed the significance of these hot spots (grey contours in Fig. 1h. FDR adjusted *P*<0.05. Also see uncorrected *P* value map in Fig. 1i). The cell detection method (either semi-automatically or manually) did not largely affect the result (Supplementary Fig. 1e,f). We performed this analysis for all the data, including layers 2/3, 4 and 5 (Supplementary Fig. 1c,d,g–l), and significant hot spots were observed in layers 2/3 in the majority of volumes (16 out of 26 volumes from 14 mice), albeit less evident in layer 4 (3 out of 14 volumes from 10 mice) and not observed in layer 5 (0 out of 10 volumes from 6 mice, Supplementary Fig. 1k,l). The radius of the centre peak was approximately 5 μm, obtained by fitting with a 2D Gaussian function to the *P* value map (Fig. 1i). The nearest peaks from the centre were often located at the distance of 15–20 μm. However, spatial patterns of the hotspots were variable across animals (Supplementary Fig. 1). These suggest that in the mouse V1, cells tend to be aligned vertically and repeatedly located in the horizontal direction with a roughly regular interval, although its spatial patterns are variable across animals.

### Similarity of orientation preference in minicolumns

**Figure 2:**
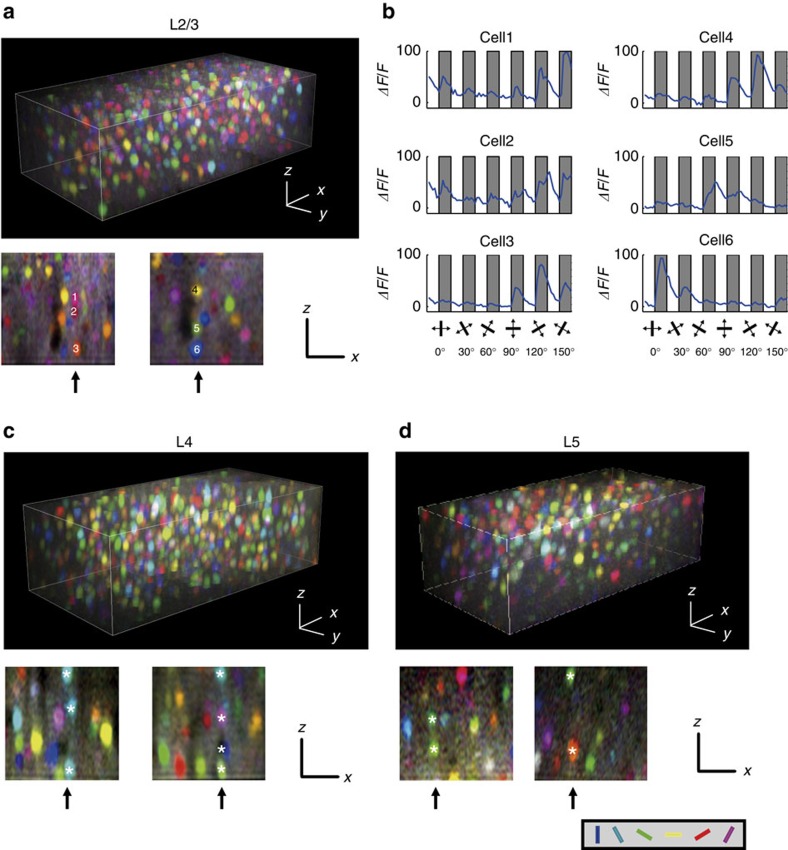
Reconstruction of three-dimensional orientation maps. (**a**,**c**,**d**) Orientation maps reconstructed for layer 2/3 (**a**), layer 4 (**c**) and layer 5 (**d**). Calcium signal changes are calculated for all the pixels, and every pixel is coloured according to the response to the orientation stimuli (hue: preferred orientation; lightness: response magnitude; saturation: gOSI). The upper panels show a three-dimensional map. The lower panels show *y*–*z* side sections (**a**,**c**,**d**). Vertically aligned cell rows are indicated by arrows and marked by numbers (1–6) or white asterisks (*). (**b**) Calcium transients of cells shown in **a** (1–6) in response to the orientation stimuli. Grey shaded: stimulation periods; white: blank periods. The angle of orientation presented during each stimulation period is illustrated at the bottom. Scale bars, 30 μm.

Thus, the existence of minicolumnar structures in the mouse V1 became evident. Next, we examined the 3D functional architecture of orientation selectivity (numbers of responsive and selective neurons are summarized in Supplementary Table 1). From the visually evoked calcium signals, we constructed colour-coded pixel-based 3D orientation maps (Fig. 2a,c and d; see Methods). In horizontal sections of the 3D maps, as was observed in previous studies^14^, we observed a salt-and-pepper distribution of neurons with different preferred orientations in layers 2/3 to 5 (Supplementary Fig. 2a–c). To examine whether any discernible vertical clusters of similarly tuned neurons exist, we resliced the 3D maps in the x–z plane (lower panels in Fig. 2a, c, and d). Three vertically arranged cells (1–3 with white letters in the lower panel of Fig. 2a) were tuned to similar orientations, which were also evident in the averaged time courses (see Methods for calculations) from the same neurons (Fig. 2b), but another set of three vertically arranged cells (4–6 in Fig. 2a) were tuned to diverse orientations. Vertically aligned responsive neurons in layers 4 and 5 were also tuned to similar orientations in some cases, but diverse orientations in other cases (lower panels in Fig. 2c,d). Therefore, we hypothesized that vertical clusters of cells with similar orientation selectivity might be embedded within an apparently intermingled functional architecture.

**Figure 3:**
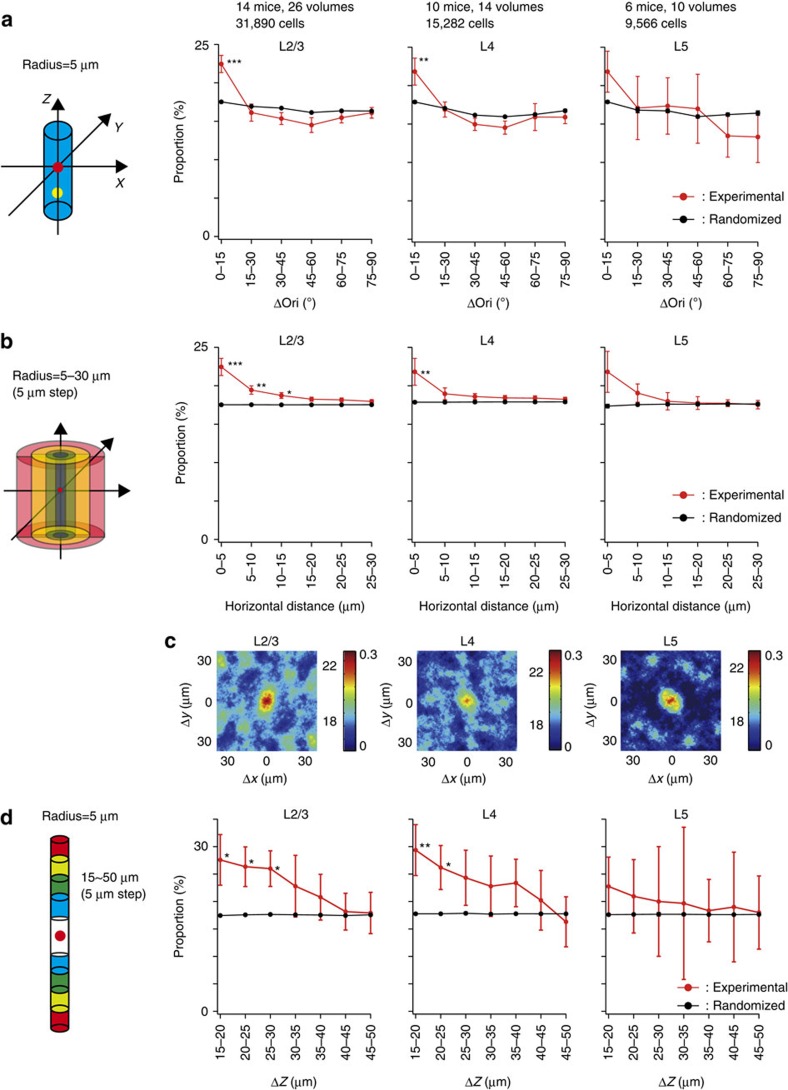
Proportion of neurons with similar orientation selectivity in a minicolumn. (**a**) The proportion of neurons with similar orientation selectivity in a minicolumn (radius=5 μm). The proportion of cell pairs is plotted as a function of Δori between cell pairs . Significant differences are observed in the Δori=0–15° bin in layer 2/3 (*P*<0.001, *n*=26 volumes from 14 mice) and in layer 4 (*P*<0.01, *n*=14 volumes from 10 mice). Red: experimental data; black: randomized data. (**b**) Horizontal distribution of cell pairs with similar orientation selectivity. The Δori between pairs of orientation-selective cells located at 0–5, 5–10, 10–15, 15–20, 20–25 or at 25–30 μm from each other in the horizontal direction is calculated and the proportion of similarly tuned cell pairs (Δori<15°) is plotted as a function of horizontal distance. ^***^P<0.001, ^**^P<0.01 and *P<0.05. The same analysis is performed for the randomized maps. Red: experimental data; black: randomized data. (**c**) The proportion map of similarly tuned cell pairs is plotted as a function of relative horizontal positions between cell pairs. The colour bar shows the proportion (left, %) and the SI (right). (**d**) Vertical distribution of similarly tuned cells. The Δori between cell pairs located within 5 μm in the horizontal direction and at 15–20, 20–25, 25–30, 30–35, 35–40, 40–45 or at 45–50 μm in the vertical direction is calculated and the proportion of similarly tuned pairs (Δori<15°) is plotted as a function of vertical distance. A significant difference is observed at 15–20, 20–25 and 25–30 μm in layer 2/3 (**d** left, *n*=26 volumes from 14 mice) and at 15–20, 20–25 μm in layer 4 (**d** middle, *n*=14 volumes from 10 mice). Red: experimental data; black: randomized data. **P<0.01, and *P<0.05.

To examine whether the vertical clusters of similarly tuned cells exist more often than expected from random distributions, we tested whether the proportion of cells with a similar orientation preference is higher within the same minicolumn. We selected vertically aligned neurons (along the entire depth of image, 100 μm) that were within 5 μm from each other (size similar to the radius of minicolumns; Fig. 1i) in the horizontal direction and regarded these neurons as belonging to the same minicolumn. We calculated the differences in preferred orientations (Δori) between all the pairs of vertically aligned orientation-selective neurons and plotted the proportion of pairs against Δori (Fig. 3a, red lines). We observed similarly tuned pairs (Δori<15°) more often than other pairs in all the layers. However, we observed that the preferred orientations of neurons in a local volume were not completely equally distributed but were biased towards some orientations^10^,^17^,^18^,^19^,^20^. Similarly tuned pairs could be found more often in such biased distributions, and the above results may have reflected this bias effect. To test this possibility, we generated 1,000 randomized orientation maps for each experiment by shuffling preferred orientations among orientation-selective cells and by preserving their 3D positions (Fig. 3a, black lines). The experimental data showed a slightly but significantly higher proportion of cell pairs than the randomized data at Δori<15° (*P*<0.001, *n*=26 volumes from 14 mice, Mann–Whitney *U*-test with the Bonferroni correction) but not other relative orientations in layer 2/3 (*P*>0.05, *n*=26 volumes from 14 mice, Mann–Whitney *U*-test with the Bonferroni correction). Similar results were observed at Δori<15° in layers 4 and 5; however, a statistically significant difference was observed only in layer 4 (*P*<0.01, *n*=14 volumes from 10 mice for layer 4; *P*=0.38, *n*=10 volumes from six mice for layer 5, Mann–Whitney *U*-test with the Bonferroni correction). Thus, vertically aligned cells within the same minicolumn tend to be similarly tuned for orientation, although the clustering is much weaker than that in other mammals that have clear orientation columns, such as cats or monkeys. We confirmed that neuropil contamination did not affect the results of functional clustering of neurons within the same minicolumns (Supplementary Fig. 3).

Following this finding, we investigated whether this tendency is limited to the same minicolumn. To this end, we plotted the proportion of similarly tuned pairs (Δori<15°) of orientation-selective cells as a function of horizontal distances between the pairs (Fig. 3b, red lines). We repeated the same analysis for the randomized maps (Fig. 3b, black lines). We observed that the similarity rapidly decreased in cell pairs at 5–10 μm in the horizontal direction, suggesting that the similarity was primarily limited to the same minicolumn. Furthermore, we defined a similarity index (SI, right axis in Fig. 3c, see Methods for the definition), which serves as an index to evaluate the tuning similarity between chosen cell pairs in experimental maps against those cell pairs in randomized maps. We made maps of SI as a function of the relative horizontal positions (Δ*x*, Δ*y*) between cell pairs (Fig. 3c, see Methods). In layers 2/3, 4 and 5, cell pairs with high SI were restricted within ∼5 μm (Fig. 3c). These results suggest that the tendency for the vertical alignment of similarly tuned cells was limited to the same minicolumn.

Further, we evaluated whether similarly tuned cells are clustered or are uniformly distributed along the vertical direction of the minicolumn. We chose all the pairs of orientation-selective cells within the same minicolumn and plotted the proportion of similarly tuned pairs (Δori<15°) as a function of the vertical distance (Δ*z*=5 μm) between the pairs (Fig. 3d). We observed a slightly but significantly higher number of similarly tuned cells in a closer vertical distance in layer 2/3 (*P*<0.05 at 15–20, 20–25 and 25–30 μm, *n*=26 volumes from 14 mice, Mann-Whitney U-test with the Bonferroni correction, Fig. 3d, left) and in layer 4 (*P*<0.01 at 15–20 μm, *P*<0.05 at 20–25 μm, *n*=14 volumes from 10 mice, Mann-Whitney U-test with the Bonferroni correction, Fig. 3d, middle). These results indicate that similarly tuned cells tend to cluster along the vertical direction (∼60 μm within 100 μm length) of a minicolumn. Our results may suggest that functional minicolumns are vertically short units restricted in a single layer^21^. However, due to the very posterior location of V1, minicolumns are not perfectly straight, but slightly curved. Although the curvature was not visually evident within 100 μm, the length of clustering might be underestimated because of the curvature.

To test whether cells belonging to the same minicolumn tend to work together, we investigated the noise-correlation during visual stimulation (Supplementary Fig. 4). We calculated the noise-correlation of neighbouring cells as a function of horizontal distances between the pairs. We repeated the same analysis for the randomized data. We observed that the noise-correlation in cell pairs belonging to the same minicolumn was significantly higher than that of randomized data and the noise-correlation decreased as the horizontal distance increased. Similar tendencies were observed in other layers, although they were not statistically significant. These results suggest that cells belonging to the same minicolumn tend to fire together at least in layer 2/3.

### Clustering of similarly tuned cells in specific minicolumns

**Figure 4:**
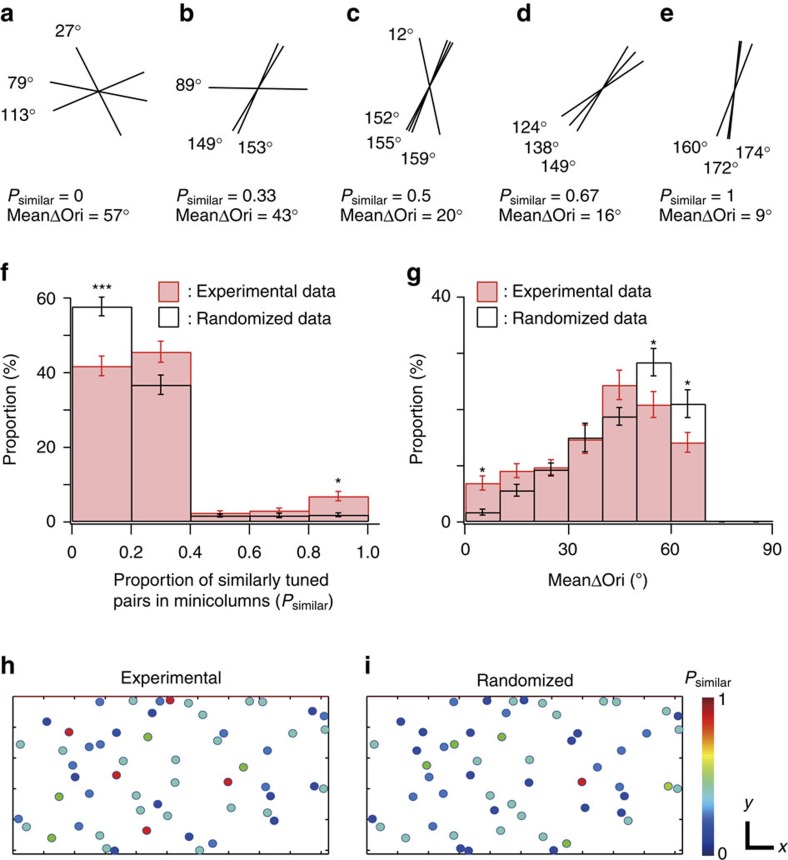
Clustering of similarly tuned cells in specific minicolumns. (**a**–**e**) Representative examples of minicolumns with various similarity. Tilting angles of black bars represent preferred orientations of individual neurons within each minicolumn. (**f**,**g**) The histograms of *P*_similar_ (**f**) and meanΔori (**g**) of minicolumns. red: experimental data, black: randomized data. *P<0.05, ^***^P<0.001, *n*=50 volumes including layers 2–5 from 30 mice, Mann–Whitney *U*-test with the Bonferroni correction. (**h**,**i**) *P*_similar_ of minicolumns in the FOV from experimental data (**h**) and randomized data (**i**). Each circle indicates the location of minicolumn and its value of *P*_similar_ with colour. Scale bars 30 μm.

We observed that neurons in the same minicolumn tended to share a similar orientation preference; however, this tendency was modest. Thus, we examined whether each minicolumn uniformly has modest similarity or whether there are a small number of minicolumns where the similarity is very high. First, we chose minicolumns that contain at least three orientation-selective cells (see Methods for the details). To assess the similarity of preferred orientations in minicolumns (Fig. 4 and Supplementary Fig. 5), we calculated two different measures; ‘proportion of similarly tuned cell pairs (Δori<15°) in individual minicolumns (*P*_similar_)', and ‘mean Δori between all cell pairs in minicolumns (meanΔori)' (Fig. 4a–e). In both measures, proportion of minicolumns with high similarity (*P*_similar_>0.8 in Fig. 4f or mean Δori<10° in Fig. 4g) were significantly higher in experimental data than that in the randomized data (*P*<0.05, *n*=50 volumes from 30 mice, Mann–Whitney *U*-test with the Bonferroni correction), indicating the clustering of similarly tuned cells in specific minicolumns. In Fig. 4h,i, minicolumns with at least three orientation-selective cells were colour-coded by their *P*_similar_ from experimental data (Fig. 4h) and from randomized data (Fig. 4i). Minicolumns with high similarity were scattered and functionally separated from neighbouring ones without a global orientation map in horizontal direction. Thus, mouse V1 is a mixture of vertical clusters of neurons with various degrees of similarity in orientation selectivity.

### 3D organization of SPF tuning and retinotopic positions

**Figure 5:**
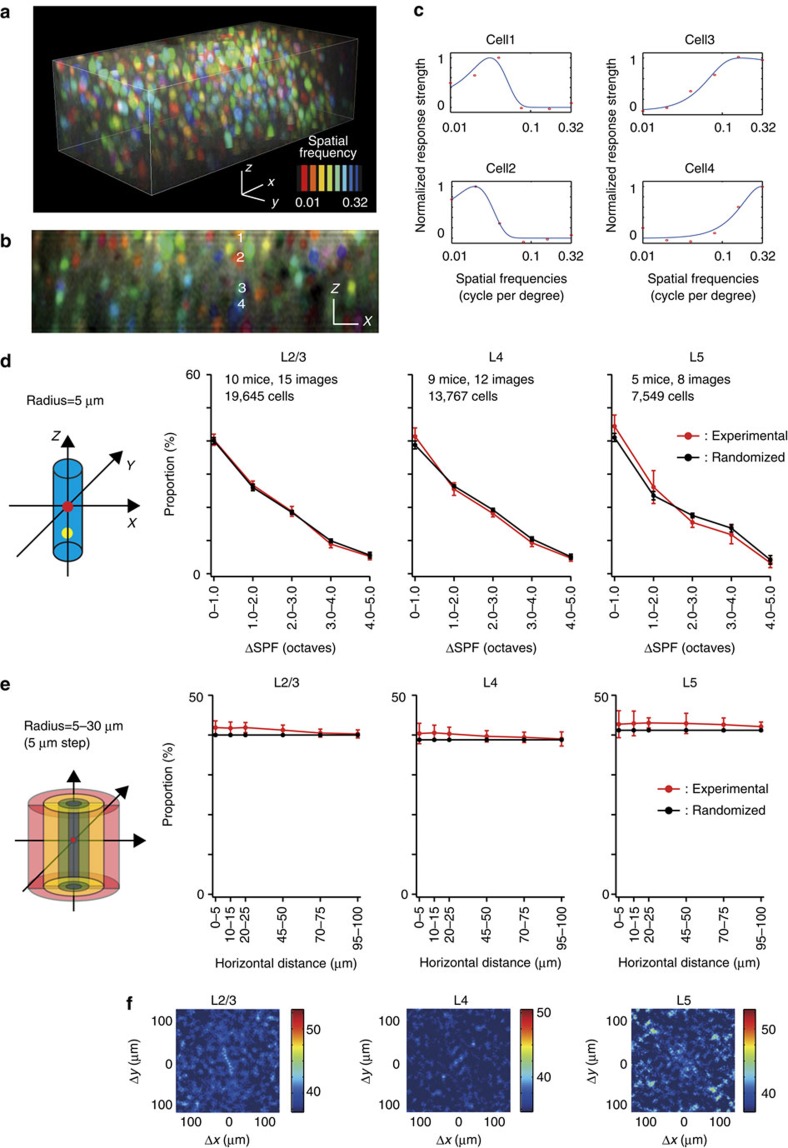
A 3D map of the SPF and the proportion of neurons with similar SPF tuning within a minicolumn. (**a**) Three-dimensional SPF tuning map. Each colour represents a SPF, as indicated in the colour bar. Scale bars, 30 μm. (**b**) A *x*–*z* vertical section of the 3D SPF map in **a**. Scale bars, 30 μm. (**c**) SPF tuning curves of neurons 1–4 in **b** are fitted with the DOG function. (**d**) The proportion of cell pairs within a minicolumn as a function of logarithmic differences between the preferred SPFs (ΔSPF) of cell pairs, obtained from experimental (red line) and randomized maps (black line). (**e**) The proportion of similarly tuned pairs (ΔSPF<1 octave) of SPF-selective cells as a function of horizontal distances between the cell pairs for experimental (red line) and randomized maps (black line). (**f**) The proportion map of cell pairs with similarly preferred SPFs as a function of relative horizontal positions between cell pairs. The colour bar shows the proportion (%).

We further examined the 3D functional architecture of other visual features, spatial frequency (SPF) tuning and retinotopic positions. SPF columns are suggested to exist in the visual cortex of higher mammals^22^,^23^,^24^. In the visual cortex of rodents, the SPF tuning of individual neurons has been systematically described^25^,^26^,^27^; however, its functional architecture has not been well described. We examined whether the vertical alignment of cells with similar SPF tuning exists in the mouse V1. The vertical sections of 3D pixel-based SPF maps (Fig. 5a) showed no clear vertical alignment of neurons with similar SPF tuning (Fig. 5b,c). To determine the preferred SPFs of neurons, their SPF tuning curves were fitted with the difference of Gaussian (DOG) function (Fig. 5c, see Methods section). The distribution of the preferred SPFs of neurons in a local volume were not uniform, but biased towards a particular SPF (Supplementary Fig. 6, Supplementary Table 2). To quantify the similarity in preferred SPFs of neurons within a minicolumn, the proportion of cell pairs within a minicolumn was plotted as a function of logarithmic differences between the preferred SPFs (ΔSPF) of cell pairs (Fig. 5d). The distribution did not differ from that obtained from randomized maps in all the layers (*P*>0.05 in all the bins in all the layers, *n*=15 in layer 2/3, *n*=12 in layer 4, and *n*=8 in layer 5; Mann–Whitney *U*-test with the Bonferroni correction), suggesting that this tendency was due to biases in the distribution of the preferred SPF. Furthermore, we examined whether there is any clustering of the SPF preference at a larger spatial scale, up to 100 μm. We plotted the proportion of similarly tuned pairs (ΔSPF<1 octave) of SPF-selective cells as a function of horizontal distances between the cell pairs for experimental and randomized maps (Fig. 5e). Even at longer distances between cell pairs, we found no significant differences between experimental and randomized maps in all the layers (*P*>0.05 in all the bins in all the layers, *n*=15 in layer 2/3, *n*=12 in layer 4 and *n*=8 in layer 5; Mann–Whitney *U*-test with the Bonferroni correction). Furthermore, the proportion maps of cell pairs with similar preferred SPFs (ΔSPF<1 octave) as a function of the relative horizontal positions between cell pairs (Fig. 5f) showed no discernible structure in layers 2/3, 4 and 5 (Fig. 5f), suggesting that there is no global SPF map with a spatial scale <100 μm in mouse V1. These results suggest that SPF tuning is completely intermingled and it is not related to minicolumns and no global SPF map exists in layers 2–5.

In contrast, retinotopic positions are coarsely clustered (Supplementary Figs 7 and 8, Supplementary Table 3). However, the scale of the clustering is much larger than that of minicolumns and likely corresponds to a global retinotopic map^28^,^29^. Altogether, functional micro-architectures for orientation, SPF, and retinotopic positions are fundamentally different, and minicolumns are weakly but specifically related to orientation selectivity but not to SPF or to retinotopic position.

### Functional architecture of microcolumns

Finally, we analysed the functional architecture of another anatomical columnar structure, microcolumn, which is a group of neurons roughly located vertically, and their apical dendrites (layer 2/3 and 5 pyramidal neurons) make a bundle in the upper layers^6^,^7^. We performed *in vivo* two-photon calcium imaging of apical shaft dendrites of layer 5 neurons^30^,^31^. Layer 5 pyramidal neurons support back-propagating action potentials into apical dendrites, and this dendrite-invading activity dominate synaptic calcium signals in dendrites *in vivo*^30^,^31^,^32^. Therefore, dendritic calcium transients reflect somatic action potentials. We investigated whether layer 5 neurons in the same microcolumn share visual selectivity by examining the visual selectivity of apical dendrites consisting of single dendritic bundles.

**Figure 6:**
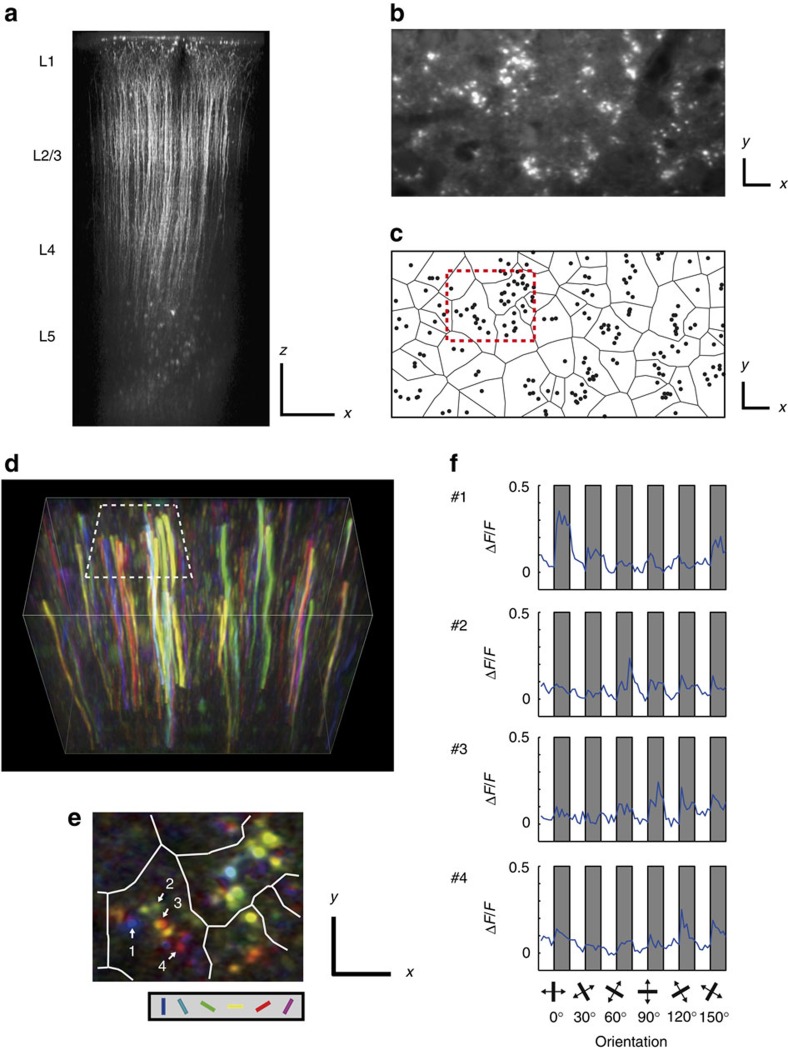
Orientation selectivity of dendrites within a dendritic bundle. (**a**) Apical dendrites and cell bodies of OGB1-AM loaded layer 5 neurons. Scale bars 100 μm. (**b**) A *x*–*y* section of apical dendrites of OGB1 loaded layer 5 neurons. Dendritic bundles can be clearly visible. Scale bars, 10 μm. (**c**) To quantify the visual selectivity of dendrites, individual dendrites are detected, and dendritic bundles are identified using Voronoi tessellation and the nearest neighbour method (see Methods). The black circles show dendrites, and each dendritic bundle is separated by black lines. The red dotted rectangle corresponds to the white dotted rectangle in **d** and is magnified in **e**. Scale bars, 10 μm. (**d**) A three-dimensional orientation map of dendrites. Every pixel is coloured according to the response to the orientation stimuli (hue: preferred orientation; lightness: response magnitude; saturation: gOSI). Each dendrite shows single orientation selectivity along the entire length in the image. Volume size is 128 μm (*x*) × 64 μm (*y*) × 100 μm (*z*). (**e**) A *x*–*y* section of orientation map of dendrites selected from a volume in **d** and the area corresponding to the white dotted rectangle in **d** is magnified. Each dendritic bundle is separated by white lines. Dendrites in the same bundle have different orientation preferences. Scale bars, 10 μm. (**f**) Averaged signal changes in dendrites #1–4 (the number is shown in **e**) in response to the orientation stimuli. Grey shaded: stimulation periods, white: blank periods. The angle of orientation presented during each stimulation period is illustrated at the bottom.

To visualize calcium signals in dendritic bundles of layer 5 pyramidal neurons, we injected OGB1-AM only into layer 5 using the bolus loading method (Fig. 6a) and imaged their apical dendrites in the middle of layer 2/3 (∼250 μm from the cortical surface) either 2D or 3D. Figure 6d,e shows pixel-based orientation maps of dendrites, suggesting that dendrites in individual bundles had different orientation preferences. To quantify the visual selectivity of dendrites, we detected apical dendrites in 2D *x*–*y* images (Fig. 6b,c, see Methods) and extracted time courses of calcium signals of the dendrites (Fig. 6f. Numbers of responsive and selective neurons are summarized in Supplementary Table 4).

Dendritic bundles were discernible in 2D *x*–*y* images (Fig. 6b) and were defined by Voronoi tessellation and by the nearest neighbour method^33^,^34^,^35^ (Fig. 6c; see Methods). After identifying the dendritic bundles, we analysed the bundles that contained at least two selectively responsive dendrites (440 bundles for orientation, 191 for SPF and 388 for the retinotopic position).

Because apical dendrites of layer 5 neurons occasionally form branches (although the branching is relatively less in the primary visual cortex of rodents^6^,^36^), some dendrites in the same bundle may belong to the same cell. We could not anatomically identify the branched dendrites from the same soma because dendrites loaded with OGB-1 AM were not bright enough to follow thin dendrites to branching points. We physiologically identified dendrites belonging to the same cell by finding highly correlated calcium signals and similar visual selectivity in pairs of dendrites (Supplementary Fig. 9; see Methods for details) and eliminated the duplicated counts. Twenty two percent (1,460 dendrites were eliminated from 6,633 selectively responded dendrites) of dendrites were eliminated by this correction.

**Figure 7:**
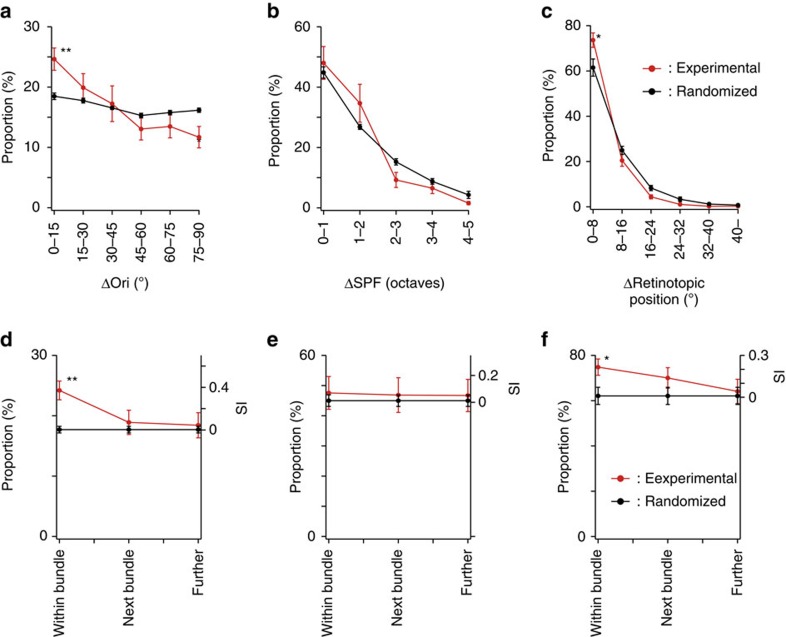
Proportion of dendrites with similar response properties within a dendritic bundle. (**a**–**c**) The proportion of dendrites is plotted as a function of Δori (**a**), ΔSPF (**b**) and Δretinotopic position (**c**) between dendrite pairs within a dendritic bundle after eliminating the duplicated counts of branched dendrites. (**d**–**f**) The proportion of similarly tuned dendrite pairs is plotted for dendrite pairs within the same bundle, between neighbouring bundles and between bundles further apart. The similarly tuned dendrite pairs are defined by Δori<15° for orientation (**d**), ΔSPF<1 octave for SPF (**e**) and Δretinotopic position<8° for retinotopic positions (**f**). ^**^P<0.01 and *P<0.05. Red: experimental data; black: randomized data.

To quantify the similarity in the visual selectivity of dendrites in the same bundles, we calculated differences in visual preferences of the pairs of dendrites within the same bundle for the orientation, SPF, and retinotopic position selectivity, and plotted the proportion of pairs of dendrites within the same bundle against the differences in visual preferences (Fig. 7a–c, The plots before eliminating the duplicated counts of dendrites are shown in Supplementary Fig. 10). Dendrites within the same bundle showed higher similarity in orientation preference (Fig. 7a, Δori=0–15°), and weak but significant differences were observed between experimental and randomized maps at Δori=0–15° and 75–90° (*P*<0.01, *n*=42 images from 16 mice, Mann–Whitney *U*-test with the Bonferroni correction for multiple comparisons).

Concerning the SPF, the similarity of dendrites within the same bundle was not different between experimental and randomized maps (Fig. 7b, *P*>0.05, *n*=25 images from 13 mice, Mann–Whitney *U*-test with the Bonferroni correction for multiple comparisons). Concerning the retinotopic position, the similarity of dendrites within the same bundle was significantly higher in experimental maps than in randomized maps (Fig. 7c, Δretinotopic position=0–8°), and significant differences were observed at Δretinotopic position=0–8° (*P*<0.05, *n*=26 images from 11 mice, Mann–Whitney *U*-test with the Bonferroni correction for multiple comparisons). These results showed that apical dendrites within the same bundle tend to be more similarly tuned for orientation selectivity and for retinotopic positions than random distribution but are not more similarly tuned for SPF.

To test whether this similarity is restricted to the same dendritic bundles or is spread to neighbouring bundles or even farther, we examined the similarity in the visual selectivity of dendrite pairs between neighbouring bundles and between bundles that were farther apart. Concerning orientation selectivity (Fig. 7d), the similarity within a bundle was significantly higher (*P*<0.01, *n*=42 images from 16 mice, Mann–Whitney *U*-test with the Bonferroni correction for multiple comparisons) than that in randomized dendrite maps (see Methods for the procedure of the randomization of dendrite maps), but the similarity in neighbouring bundles and bundles that were farther apart was not significantly different. Concerning the SPF (Fig. 7e), the similarity in the same bundles, neighbouring bundles, and bundles that were farther apart was not significantly different from that in the randomized dendrite maps. Concerning retinotopic positions (Fig. 7f), the similarity in the neighbouring bundles was less than that in the same bundle but still higher than that in randomized dendrite maps, albeit not statistically significant.

In summary, we found that clustering of neurons with similar orientation preferences is limited to the same microcolumn, the SPF is not related to microcolumns, and the clustering of neurons with similar retinotopic positions is larger than microcolumns, all resembling the functional architecture of minicolumns.

## Discussion

In this study, we examined functional organization of minicolumns and microcolumns, which are anatomical units of cortical architecture in the mouse V1. First, we showed that anatomically minicolumnar structures exist in the mouse V1. The radius of anatomical minicolumns was ∼5 μm that is similar to the previously reported half width of minicolumns in the monkey striate cortex^5^ (5–10 μm). The distance between adjacent minicolumns was 15–20 μm; this value is also comparable with that in the monkey striate cortex^5^ (∼18.5 μm: which was calculated from 54 crossings of minicolumns along a 1 mm length) and with that in human^4^ (24 μm), although the spacing has sometimes been believed to be wider possibly due to confusion with microcolumns (30–50 μm).

Next, we found that neurons with similar orientation tuning were weakly but significantly clustered in a short vertical segment of minicolumns and the clustering was limited to the same minicolumn. The SPF selectivity was completely intermingled. The retinotopic position maps were scattered but coarsely clustered, likely corresponding to a global retinotopic map. Altogether, functional microarchitectures for these three visual features are fundamentally different, and minicolumns and microcolumns are specifically related to orientation selectivity but not to SPF or to retinotopic position. We also examined the functional architecture of microcolumns, another anatomical units of cortical architecture, and obtained basically the same conclusions as those in minicolumns.

Thus, we found that three different visual functions, orientation, SPF and retinotopic position selectivity, showed different functional micro-architectures. Why these architectures are different could be an important question to understand the visual processing in V1. Transformations of selectivity from the thalamus to the cortex are suggested to be different across visual features. The cortical retinotopic position and SPF selectivity are primarily inherited from the LGN. In contrast, it is suggested that most of the orientation selectivity is formed in the cortex (see refs 37, 38, 39, but see refs 19, 40, 41), because a small number of orientation selective neurons (∼10%) localized in a specific part of LGN^42^,^43^ project mainly to layer 1 avoiding layer 4 (ref. 39). Although the precise mechanisms to generate orientation selectivity remain not fully understood^44^, the cortical minicolumnar structure may be necessary for the transformation of the selectivity. The SPF and retinotopic position selectivity may not require the minicolumnar structure because these functions are primarily coming from the LGN.

**Figure 8:**
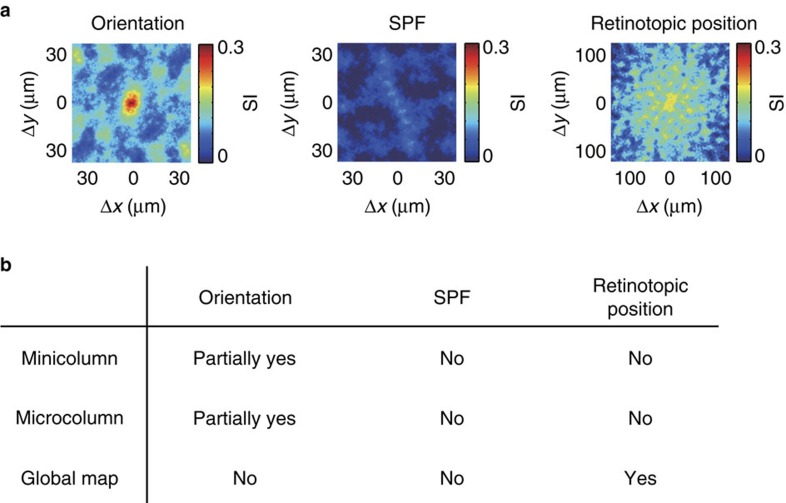
Summary. (**a**) Maps of similarity index (SI, see Results and Methods sections for detail) for orientation, SPF and retinotopic position from layer 2/3. For orientation, high SI is restricted in the centre, suggesting that the vertical clustering of similarly tuned cells is limited to the same minicolumn. (**b**) Presence or absence of the functional clustering for three visual functions (orientation, SPF, retinotopic position), in relation to minicolumn or microcolumn, or as a global map in the mouse V1.

Further, we found that the vertical clustering was found only in a part of minicolumns, and others were composed of neurons with a variety of orientation preferences. Thus, we suggest that the mouse V1 is a mixture of vertical clusters of neurons with various degrees of similarity in orientation selectivity (Fig. 8). Anatomical minicolumns have been also reported in cats and monkeys^5^ but these animals have the orientation columns, whose width is much larger than that of minicolumns. In the orientation columns of cat visual cortex, nearly 100% of cells have a similar orientation preference^14^,^45^. Therefore, the clustering in mouse visual cortex is much weaker than that of the animals with orientation columns.

We found orientation-tuned neurons are aligned vertically in short length. It has been suggested that minicolumns and microcolumns are candidates of fundamental functional units for cortical processing^1^ and may serve as output units^35^. Further, each layer has different and specific input and output targets^46^. Thus, cortical output units may be the short vertical clusters of neurons, which carry various (similarly or diversely tuned) orientation information^47^,^48^.

How do the similarly tuned minicolumns emerge? Several possible mechanisms can be considered. (1) These minicolumns may be composed of specific neuronal subtypes (for example, morphological, physiological or projection types), although classification of layer 2/3 excitatory neurons have not been well elucidated. The minicolumnar anatomical arrangement of different projection subtypes of layer 5 neurons^21^ may support this idea. (2) Minicolumns with similarly selective neurons may arise from common progenitor cells. Neurons from a common progenitor (clonal cells) distribute sparsely in several minicolumns in the mouse cerebral cortex^49^. Although the pattern of the distribution of the clonal cells has not been fully analysed, some minicolumns may be composed of neurons with more homogenous clonal origin, and others may be composed of neurons with more heterogeneous origins. It has been suggested neurons that originated from a single clone tend to share similar orientation tuning in mouse^50^,^51^. (3) Minicolumns with similarly selective neurons may be remaining of an initial bias. There is a strong bias in the distribution of orientation preferences at shortly after eye opening; however, this bias reduces thereafter^20^,^52^. Thus, the proportion of similarly tuned neurons is higher after eye opening, whereas this proportion is reduced on the reduction of the bias. If the reduction of the bias occurs with varying degrees between minicolumns, then some minicolumns remain biased even after development, and these minicolumns may correspond to minicolumns with similar orientation-tuned neurons.

We found that the mouse V1 is a mixture of vertical clusters of neurons with various degrees of similarity in orientation selectivity (Fig. 8). On the other hand, higher mammals like monkeys and cats have larger columnar organization with similar orientation-selective neurons^5^,^53^, even at single-cell level^45^. Therefore, the functional organization of orientation-selective neurons in mouse V1 is not just the reduced version of higher mammals. Vertical clustering of neurons with similar orientation selectivity in mouse may correspond to the orientation macro-columns in higher mammals, and may contribute to send information of a single orientation. On the other hand, minicolumns with diverse orientation selectivity in mouse has no counterpart in higher mammals. We speculate that these minicolumns with diverse orientations may be assigned to represent visual information more efficiently with a small number of neurons in the small brain. The mixed functional architecture in mouse V1 may be the compromise between the brain size and keeping the vertical clusters of similarly tuned neurons at least in a subset of clusters. Alternatively, the difference in the functional organization between mouse and higher mammals may not be due to the difference in the size of visual cortex^54^,^55^, but may be due to the difference in the developmental processes^51^,^56^,^57^.

## Methods

### Animal preparations for imaging

All experimental procedures used in this study were approved by the Animal Care and Use Committee of Kyushu University.

Experiments were performed with C57BL/6 male mice between 8 and 10 weeks old (SLC, Japan). Mice were anaesthetized with isoflurane (vapourized by a 1:1 mixture of air and O_2_, 1.0–1.5% during surgery) delivered via a small nose cone. The primary visual cortex (V1) was identified stereotaxically, and a small craniotomy (3 mm of diameter circle) was made over V1 area. The calcium indicator was loaded using the multiple cell bolus loading method. Fifty micrograms of the AM form of the calcium-sensitive dye Oregon Green BAPTA-1 (OGB-1 AM, Molecular Probes) was dissolved in 4 μl DMSO containing 20% Pluronic F-127, and then 35 μl modified ringer solution (140 mM NaCl, 2.5 mM KCl and 10 mM HEPES, pH 7.4) and 1 μl sulforhodamine 101 (SR-101, Molecular Probes, 1 mM) were added. The dissolved dye was filtered by centrifugation (Ultrafree-MC, 0.45 μm pore size, Millipore). Dyes were pressure-ejected (2–10 psi, 50 ms–5 s duration, 10 s interval, 3–10 min long) to the monocular region of the left hemisphere at the depth of 300–600 μm below the cortical surface using a glass pipette (3–5 μm tip diameter). For dendrite imaging, we injected OGB-1 AM only into layer 5 (ca. 550 μm below the cortical surface). The craniotomy was filled with modified ringer solution, sealed with a glass cover slip (5 mm diameter) and immobilized using dental acrylic.

### Two-photon calcium imaging *in vivo*

The activities of cortical neurons and dendrites were recorded three-dimensionally using two-photon microscopy (A1RMP, Nikon, Japan) with a × 25 water immersion objective lens (CFI Apo LWD 25XW, numerical aperture (NA)=1.10, Nikon or XLNPlan N × 25, NA=1.05, Olympus). A resonant scan mirror and a galvanometric scan mirror were used for x-scanning and for y-scanning, respectively. The 3D image was 512 × 256 × 35 voxels (cell body imaging: 0.5–1.0 μm per pixel, 3 μm of z-step size, dendrite imaging: 0.25 μm per pixel, 3 μm of z-step size) and scanned at 1.25–1.6 Hz. Approximately 1,000 neurons (between 700 and 3,000 cells depending on the image size) were detected within this volume (Fig. 2a,c and d). Some dendrite images were obtained two-dimensionally with 512 × 512 pixels (0.6 μm per pixel) at 4 Hz using galvanometric scan mirrors (LSM7MP, Zeiss, Germany) and a × 25 water immersion objective lens (XLNPlan N 25X, NA=1.05, Olympus). A pulsed laser was tuned at a 100-fs pulse of 80 MHz at 920 nm using a Ti:sapphire laser system (Mai Tai eHP DeepSee, Spectra-Physics). Recordings were performed between layers 2/3 and 5, and the data were collected and analysed according to the layers, which were assigned by the depth (in μm) as follows: layer 2/3, 100–350; layer 4, 350–450; and layer 5, 450–650 (ref. 58). The A–P axis was set parallel to the sagittal suture and the M–L axis was set orthogonal to the A–P axis and parallel to the imaging plane. During the recordings, mice were anaesthetized with isoflurane (0.5–1.0%), and the depth of anaesthesia was monitored and adjusted by heart rate (8–9 Hz). Body temperature was maintained using a feedback-controlled heat pad (DC Temperature Controller, FHC). Mouse eyes were immersed with silicon oil (3,000 CS) during experiments to prevent dry eyes.

### Visual stimulation

The visual stimuli were generated by a custom written program (Visual Basic) and presented on a 19 inch LCD display (60 Hz refresh rate) placed 18 cm from the right eye, spanning ∼93° (azimuth) × ∼80° (elevation) of visual space. We tested three different visual functions to investigate the functional organization: orientation, SPF and retinotopic position. The orientation tuning was studied using square-wave drifting gratings at six different orientations (30° apart) moving in two opposite directions (direction selectivity was not investigated in this study). These six orientation patterns were changed sequentially, and each orientation was presented for ∼5 s, interspersed with grey blank (uniform) stimuli of the same duration. Spatial and temporal frequencies were set at 0.04 cycles per degree (c.p.d.) and at 2 Hz, respectively. We have previously shown that properties of orientation selectivity obtained with a fixed stimulus sequence were not significantly different from those obtained with randomized one^20^. For the SPF preference analysis, drifting sinusoidal-wave gratings with six different SPFs (0.01, 0.02, 0.04, 0.08, 0.16 and 0.32 c.p.d.) were presented for ∼6 s, interspersed with grey blank (uniform) stimuli of the same duration. Drifting gratings were moved in eight directions for each SPF, and the temporal frequency was set at 2 Hz. For the retinotopic position analysis, the display around the centre was divided into 16 segments (4 × 4 grids, 16° × 16° of visual space for each segment, close to the average receptive field diameter of a single neuron in the mouse primary visual cortex^12^, and square-wave drifting gratings were presented for ∼6 s in each segment, interspersed with grey blank (uniform) stimuli of the same duration. We used a relatively large grid size because of the difficulty in obtaining responses using a smaller size. Drifting gratings were moved in eight directions for each segment, and spatial and temporal frequencies were fixed at 0.04 c.p.d. and at 2 Hz, respectively. Stimulation protocols started with blank (uniform mean luminance), followed by visual stimulation. The presentation of stimuli was ordered and repeated 20 times for orientation and for SPF and 10 times for the retinotopic position analysis.

### Data analysis for calcium imaging of soma

The analysis of data was performed with Matlab (Mathworks). Three-dimensional volume data were aligned based on the correlation method using the discrete Fourier transform phase. The realigned data were used for all the analyses hereafter. We performed two different analyses, pixel-based and cell-based analyses for orientation and SPF tuning analyses. The retinotopic position was analysed only using the cell-based method.

Cell-based analyses were performed for the quantitative investigations. Imaged cells were automatically recognized by template matching using convolution mask images. Over 98% of cells were recognized by this method. The rest of unrecognized cells were manually selected by eyes. The automatic cell-recognition sometimes double-counted a single cell, therefore, we manually eliminated duplicated cells in all the 3D frames by investigating each frame using our eyes. The centroid of cells was determined from a mask image of cells. The position of each cell in the 3D volume was registered as a *xyz* coordinate of its centroid.

Time courses of fluorescent change were extracted by averaging a circle of ∼3 μm radius around the centroid and normalized by the baseline fluorescence (Δ*F*/*F*). Baseline fluorescence was obtained during 2 volume recording just prior to the visual stimulation. Out-of-focus fluorescent contamination (neuropil contamination) was corrected by subtracting the averaged fluorescence signals of the outer circle of the mask image. We created a circular ring around the cell mask and constructed a neuropil mask ring. This neuropil mask ring had a ring width (distance between the inner and outer radii of a ring) of ∼3 μm and was placed ∼2 μm apart from the edge of the cell mask to reduce the possible overlap between the ring and cell. If the neuropil mask was overlapped with the neighbouring cell mask, the overlapped area was removed from neuropil mask. The time course of neuropil signal was calculated by averaging the signal changes in the neuropil mask. The neuropil signal was subtracted from the fluorescence signal of neurons (contamination ratio=0.3; see ref. 27). Because we used few pixels around the centre of the cell for the fluorescence signal calculation, we could set the contamination ratio relatively small and constant for all the cells.

### Data analysis for visual response and selectivity

The *P* value for responsiveness was obtained from analysis of variance (ANOVA) across blank and stimulus periods, and the *P* value for selectivity was obtained from ANOVA across stimulus periods.

The preferred orientation was calculated using vector averaging^59^, defined by the following equations:


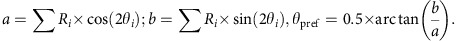


*R*_*i*_ is the response to the *i*th orientation *θ*_*i*_ (6 orientations, 30° apart, spanning 0–150°). *θ*_pref_ is the preferred orientation. As an index of orientation selectivity, the orientation index (OI) was calculated using the following formula:


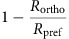



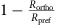
 where *R*_pref_ is the response to the preferred orientation, and *R*_ortho_ is the response to the orthogonal orientation to the preferred orientation. gOSI (global OSI), which is equivalent to 1-circular variance (CV), was also calculated using the following formula:


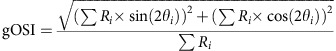


where *R*_*i*_ is the response to the *i*th direction *θ*_*i*_ (ref. 60). Neurons and dendrites were considered responsive when they met criteria that the *P* value for responsiveness was <0.001 and the maximum response was more than 3%. Among responsive neurons and dendrites, sharply orientation-selective ones were defined when they met criteria that the *P* value for selectiveness was <0.001 and the OI was more than 0.5.

The responses to different SPFs were fitted with the DOG function^61^. The preferred SPFs of neurons and dendrites were determined from the peak of the DOG fitting curve. DOG fittings were evaluated by the *R*-squared values. As an index of SPF selectivity, the selectivity index (SEI) was calculated using the following formula:


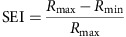


where *R*_max_ is the maximum response and *R*_min_ is the minimum response. Neurons and dendrites were considered responsive when they met criteria that the *P* value for responsiveness was <0.001 and the maximum response was more than 3%. Among responsive neurons or dendrites, SPF selective ones were defined when they met criteria that *P* value for selectiveness was <0.001, the SEI was more than 0.5, and the *R*-squared value was more than 0.7. Only the selectively responsive neurons and dendrites were used for the further analysis.

The pixel-based analysis was performed to construct the orientation and SPF maps. To obtain an orientation map, calcium signal changes were calculated for all the pixels, and every pixel was coloured according to the response to the orientation or SPF stimulation (hue: preferred orientation or SPF, lightness: response magnitude, and saturation: gOSI or SEI). The map shows pixels that were activated by specific orientation or SPF by the different colours and white colour indicates pixels that were responsive but unselective.

### Analysis for the spatial pattern of cell positions

Let (*x*_*i*_, *y*_*i*_, *z*_*i*_) be the position of *i*th cell (*i*=1, ..., *N*).

Then, relative position between *i*th cell and *j*th cell is (*x*_*j*_−*x*_*i*_, *y*_*j*_−*y*_*i*_, *z*_*j*_−*z*_*i*_).

Histogram of relative positions of any pairs of cells (in both directions) can be obtained as a 3D histogram of (*x*_*j*_−*x*_*i*_, *y*_*j*_−*y*_*i*_, *z*_*j*_−*z*_*i*_) (*i*=1, ... *N*, *j*=1, ..., *N*, *i*≠*j*)

The 3D histogram histΔ(*x*, *y*, *z*) can be represented by using Dirac's delta function *δ*(*x*),


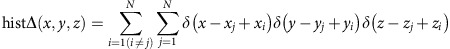


This histogram of relative cell positions is equivalent to autocorrelation of cell positions, except for the origin. Below is the proof. The function that represents positions of all the cells can be defined as:


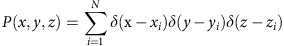


Autocorrelation of *P* (*x*, *y*, *z*) can be calculated as


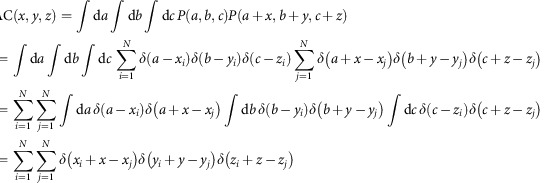


Except for the origin(*x*, *y*, *z*)=(0, 0, 0), this is equivalent to


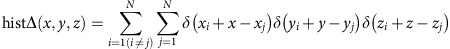


This holds for histogram of relative cell positions as a function of horizontal displacement between cell pairs, ignoring vertical displacement.


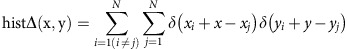


and this is exactly the same as the 2D autocorrelation of cell positions as a function of the horizontal displacement.

After smoothing with a kernel of a ball of 5 μm in radius (*f*(*x*, *y*, *z*) is 1 if |(*x*, *y*, *z*)|<5 μm, or is 0 otherwise) and dividing by the volume of the 5 μm sphere ((500*π*)/(3)) and the number of cells (*N*), the 3D histogram of relative cell positions can be converted to probability density map of neighbouring cells (probΔ(*x*, *y*, *z*)), which represents probability of finding a cell at (*x*, *y*, *z*) when one cell locates at the origin,


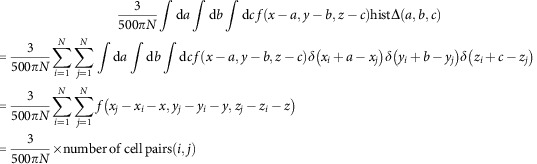


which satisfy |(*x*_*j*_−*x*_*i*_−*x*, *y*_*j*_−*y*_*i*_−*y*, *z*_*j*_−*z*_*i*_−*z*)|<5 μm.

However, when (*x*, *y*, *z*) deviates from zero, the search volume for finding the cell pairs decreases from *XYZ* to (*X*−|*x*|)(*Y*−|*y*|)(*Z*−|*z*|) ((*X*, *Y*, *Z*) is the size of the volume of FOV).

Thus, autocorrelation map has a bias that values (that is, number of cell pairs) decrease as (*x*, *y*, z) deviates from zero.

This bias is corrected by multiplying


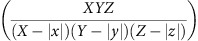


The final formula for the probability density map is:


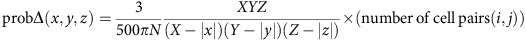


which satisfy |(*x_j_*−*x_i_*−x, *y_j_*−*y_i_*−y, *z_j_*−*z_i_*−z|)<5μm.

Similarly, the 2D histogram of relative cell positions can be also converted to probability density map of neighbouring cells probΔ(*x*, *y*), which represents probability of finding a cell at (*x*, *y*), ignoring *z* position, when one cell locates at the origin.


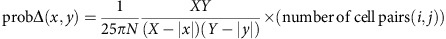


which satisfy |(*x*_*j*_−*x*_*i*_−*x*, *y*_*j*_−*y*_*i*_−*y*)|<5 μm.

### Statistics of probability density map of neighbouring cells

In the statistics of probability density map of cell location obtained from Nissl-stained image, we computed new 20,000 probability density maps from randomized data sets in which cells were randomly located in 2D space while number of cells and the space size were maintained. In the randomization of cell's location, we added one assumption to avoid overlapping the cell locations; distance between two cells was more than 5 μm. This assumption was based on both real data (see probability density map in Fig. 1b) and the literature^21^. In each pixel of probΔ(*x*, *z*), *P* value was defined as *P*=1−*j*/100 when the pixel value of probΔ(*x*, *z*) is *j*th percentile of the distribution from 20,000 randomized probability density maps. To avoid highly strict *P* value correction for multiple comparisons across many pixels (70 × 190 pixels), we used FDR controlling method^62^, rather than family-wise error rate such as Bonferroni correction. FDR method controls the expected proportion of false positive pixels among significant pixels. *P* values were adjusted under the control of FDR level at 0.05 (FDR adjusted *P* value, ref. 63). Significant level of FDR-adjusted *P* was set at 0.05. This means that 5% of significant area in a probΔ(*x*, *z*) is falsely detected as significant. For the purpose of comparison, uncorrected *P* value maps are also shown in the figure (Fig. 1b).

In the statistics of probability density map of cell location obtained by two-photon imaging (Fig. 1h,i and Supplementary Fig. 1), almost same procedure was used with slight modifications. For each imaged volume, 12,000 probability density maps were generated from randomized data set. In the randomization of cell position, another assumption was added in addition to the minimum distance between cells (>5 μm); cells should be apart more than 15 μm along *z*-direction, if the two cells were aligned vertically and located within 5 μm in *x*–*y* direction. This was based on the observation that vertically aligned cells tended to be separated ∼15 μm in two-photon imaging data (Fig. 1g). Other calculations were the same to the Nissl image analysis.

### Data analysis of Nissl-stained section

A mouse was fixed by perfusing 4% paraformaldehyde solution via vasculature system through heart. Brain was removed and post-fixed in the same fixative solution for overnight at 4°. Coronal slice (50 μm thickness) from primary visual cortex was prepared by a vibratome. Nissl staining of brain slices was performed by conventional method. After taking the photograph, Nissl-stained cells were manually detected and cell positions were registered as *x* and *y* coordinates by using the hand-made Matlab program. Probability density map was calculated as described in the previous subsection. For the quantitative measurement, the shrinkage of slice by the fixative was corrected by 15% (ref. 64).

### Data analysis for anatomical structures of minicolumns

Although we carefully defined the *z*-axis perpendicular to the cortical surface, a slight inclination of the volume persisted. To determine the vertical alignment, we constructed probability density maps (probΔ(*x*, *y*, *z*), see previous section) of neighbouring cells (Fig. 1d). The peaks of the probability density map along the *z*-axis showed the positions of vertically aligned neighbour cells. The peaks were slightly tilted from the *z*-axis (the red dotted line in Fig. 1f,g), from which the tilt of the volume data was estimated, and the tilt of the volume was corrected for all the volume data. This correction was crucial for the analysis of narrow vertical structures, such as minicolumns.

After correcting the tilt, 2D probability density map (probΔ(*x*, *y*)) of neighbouring cells as a function of the horizontal displacement between cell pairs, ignoring vertical displacement, was calculated as described in the previous section. The 2D probability density map (probΔ(*x*, *y*)) was calculated for all the cells (Supplementary Fig. 1a) or all the selectively responding cells (Supplementary Fig. 1c).

### 2D proportion map of similarly tuned cells

Similarly tuned cells were defined by setting a threshold for each visual function, 15° for the orientation, 1 octave for the SPF and 8° for the retinotopic position. 2D probability density map of similarly tuned cells was calculated as a function of the horizontal displacement from all the selectively responsive cells, ignoring the depth direction.


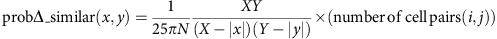


which satisfied |(*x*_*j*_−*x*_*i*_−*x*, *y*_*j*_−*y*_*i*_−*y*)|<5 μm and Δ(response selectivity)<threshold.

The 2D proportion map of similarly tuned cells (proportion_similar (*x*, *y*)) was obtained as a ratio map between a probability density map of similarly tuned cells (probΔ_similar (*x*, *y*)) and that of all the selective cells (probΔ (*x*, *y*)).


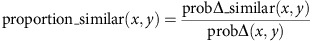


The value in 2D proportion map at (*x*, *y*) represents proportion of finding similarly tuned cells among selectively responding cells at (*x*, *y*), ignoring *z*, when one cell locates at the origin.

We defined the SI using the following formula:


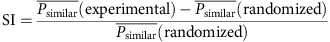


where 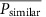 (experimental) is the proportion of cell pairs with similar response selectivity in the experimental data, and 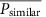
(randomized) is the proportion of cell pairs with similar response selectivity in the randomized data (see next section for the randomization procedure). SI is a useful index to directly compare the similarity across the data in the different layers and different visual selectivity.

### Data analysis for functional organization of minicolumns

The distances between cell positions and the differences in the preferred orientation, preferred SPF, or retinotopic position selectivity were calculated between all the cell pairs. To define the cells in a minicolumn, first, we chose one cell in an image. Then, the cells whose distance was <5 μm from the chosen cell were regarded as the cells in a same minicolumn. The proportion of cells with similar selectivity (Δori<15°, ΔSPF<1 octave, Δretinotopic position<8°) within a minicolumn (radius=5 μm) was calculated for each visual function. We repeated this calculation for all the cells in an image. There is a possibility that a cell can belong to more than one minicolumn.

To analyse the horizontal distribution of similarly tuned cells, we chose pairs of orientation-selective cells that were at 0–5, 5–10, 10–15, 15–20, 20–25 or at 25–30 μm (orientation) or at 0–5, 10–15, 20–25, 45–50, 70–75 or at 95–100 μm (SPF and retinotopic position) from each other in the horizontal direction and calculated the proportion of cells with similar response properties (Δori<15°, ΔSPF<1 octave, Δretinotopic position<8°). To compare the experimental results with randomness, we generated 1,000 new maps (randomized maps) for each experiment. These randomized maps contained the same proportion of orientation, SPF and retinotopic positions, and the single features were repositioned randomly in each analysis, while preserving the original 3D cell positions. From these randomly repositioned maps, we performed the same analysis as described above. We used these randomized maps for all the other analyses of randomness.

To calculate the vertical distribution of similarly tuned cells, we chose cell pairs locate within 5 μm (distance between centroids) in the horizontal direction and at 15–20, 20–25, 25–30, 30–35, 35–40, 40–45 or at 45–50 μm in the vertical direction and calculated the proportion of similarly tuned cells (Δori<15°, ΔSPF<1 octave, Δretinotopic position<8°). To compare with randomness, we used the same randomized map that was used for the horizontal distribution analysis.

### Calculation of noise correlation

Noise correlation was computed using mean Δ*F*/*F*s during stimulation of single trials. In each neuron, single trial Δ*F*/*F*s were subtracted by trial average of Δ*F*/*F* in each orientation condition. Then these values were transformed to *z*-score in each orientation. These *z*-scores were collected across orientations, resulting in 20 trials × 6 orientations (=120) data points for each cell. Pearson's correlation of these 120 data points between two cells was computed as noise correlation.

### Data analysis for calcium imaging of dendrites

We performed both pixel- and dendrite-based analyses for dendritic bundles. The pixel-based analysis was performed as described in the previous pixel-based analysis section in the minicolumn analysis. For the dendrite-based analysis, we detected all the apical dendrites on the *xy* plane images and determined their coordinates (centre pixel of the dendrite). To identify the dendrite, first, the *xy*-plane image was filtered using DOG functions. Then, the position of the local maxima of the filtered image was detected and averaged with its immediate neighbours (3 × 3 pixel squares). We regarded the pixel position as the potential centre of the dendrite when the averaged value was more than the threshold. The threshold was set as the s.d. of the DOG filtered image intensity.

Time courses of fluorescent change were extracted by averaging a circle of 5 pixels in diameter around the centre of the dendrite (smaller than the diameter of the dendrite). Visually evoked fluorescent changes were calculated as the change in fluorescence normalized by the baseline fluorescence (Δ*F*/*F*). The orientation, SPF and retinotopic position preference were calculated as described in the cell-based analysis section for minicolumns. The statistical analyses of responsiveness and the selectivity of responses were also performed as described in the cell-based analysis section for minicolumns.

### Elimination of dendrites originated from the same neurons

To eliminate dendrites that potentially originated from the same neurons, we applied the shuffle-corrected cross-correlation method^65^. First, we calculated trial averaged time-course data for each stimulus, as the shuffle-predictor of visual response. The shuffle-corrected time-course data were obtained by subtracting the shuffle-predictor from the original time-course data for each stimulus. The shuffle-corrected cross-correlogram was obtained as the cross-correlogram between shuffle-corrected time-courses. This procedure is equivalent to repeat the shuffle correction for every possible shuffles^65^. The correlation coefficients (values of the shuffle-corrected cross-correlogram at time zero) were used to determine the dendrites from the same origin.

To determine the threshold to eliminate the dendrites from the same origin, we calculated the correlation coefficients between the same and different dendrites. We chose adjacent frames from 3D images, and correlation coefficients at time zero were calculated between the same dendrite pair and the different dendrite pair using the shuffle-corrected time-course data (Supplementary Fig. 9a). Cross-correlograms between the same dendrite pair showed a sharp positive peak at time zero (Supplementary Fig. 9b), whereas between the different dendrite pair, the peak was almost absent (Supplementary Fig. 9b). The distribution of correlation coefficients at time zero from the same and different dendrite pairs was clearly separated (Supplementary Fig. 9c). We also plotted the Δori values versus correlation coefficients and observed that some of the dendrites with above threshold correlation coefficients had large Δori values, indicating that these dendrites were possibly different dendrites (data not shown). We plotted the proportion of Δori values from the same and different dendrites and set the second threshold with Δori values (Supplementary Fig. 9d). We determined the threshold of correlation coefficient and Δori values as 0.15° and 15°, respectively (Supplementary Fig. 9c,d). We calculated the correlation coefficients at time zero between all the pairs within previously defined dendritic bundles. One of the examples from Fig. 6 is shown in Supplementary Fig. 9e–h. Three dendrites with similar orientation preferences were selected from one dendritic bundle (Supplementary Fig. 9e,f). Shuffle-corrected time-courses were generated, and cross-correlograms were calculated (Supplementary Fig. 9g). The cross-correlogram between some pairs showed a strong positive peak at time zero (Supplementary Fig. 9g, right) but not between other pairs (Supplementary Fig. 9g, left). We eliminated dendrite pairs whose correlation coefficients exceeded the thresholds (Supplementary Fig. 9h). We performed a similar analysis for the SPF and for retinotopic positions and determined the threshold of correlation coefficients and the SPF or the retinotopic position as 0.15 and 1.0 octave or 8°, respectively, and eliminated dendrites that potentially originated from identical neurons (data not shown). Using these thresholds, we eliminated dendrite pairs whose correlation coefficients exceeded these thresholds. After this correction, we re-calculated the functional similarity of dendrites within a bundle as described above. The estimated proportion of duplicated dendrites (22%) originated from the same layer 5 pyramidal cells is not low from the following reasons. Past studies^36^,^66^ indicate that the bifurcation of apical dendrite of layer 5 neurons in the mouse primary visual cortex is typically observed at ∼120 μm (close to the border between layers 1 and 2/3) and much less below ∼250 μm (our analysed imaging depth). The bifurcation below ∼250 μm is mostly observed in a certain cell type, corticotectal cell (∼30% to the total population, Supplementary Fig. 1 in ref. 66). Furthermore, a study^6^ indicates that the bifurcated dendrites sometimes laterally shift to the neighbouring bundles in the rat visual cortex. Therefore, at ∼250 μm, the proportion of the bifurcated apical dendrites within the same bundle would be <30%. Thus, the estimated proportion of bifurcated dendrites (22%) is close to what we should expect from anatomy.

### Data analysis for microcolumns

Dendritic bundles in 2D *x*–*y* images were defined by Voronoi tessellation and by the nearest neighbour method. The Voronoi tessellation method was applied to assign polygons to each apical dendrite by joining the midpoints of the segments connecting the apical dendrite to its neighbours. We measured the nearest neighbour distance between two dendrites and considered dendrites in the same bundle when the nearest neighbour distance fell in the range of 1–6 μm. Then, we eliminated the demarcation line of polygons between two dendrites, and each cluster of dendrites was outlined by single polygon. We regarded clusters of at least three dendrites as dendritic bundles, according to previous studies^33^,^34^. We obtained similar size bundles (diameter of each dendritic bundle was ∼30 μm) with similar numbers of dendrite (varied between 3 and 15, 6 dendrites on average) within the bundle, as reported previously in rodents^6^,^7^,^33^,^34^.

To calculate the proportion of dendrites that share similar response selectivity within a bundle, first, dendrites to analyse were selected using the above criteria. The differences in the preferred orientation, preferred SPF and retinotopic position were calculated between all the pairs within a bundle. Then, we calculated the proportion of dendrites that share response selectivity within a bundle. To compare the experimental results with randomness, we generated 1,000 new maps for each experiment. These new maps used the same proportion of preferred orientation, preferred SPF and retinotopic positions, and the single features were repositioned randomly in each analysis while preserving the original dendrite positions. From these randomly repositioned maps, we performed the same analysis as for the experimental data.

### Statistical analysis

All the data were presented as mean± s.e.m., unless stated otherwise. We did not exclude any data point. Either two-sided Mann–Whitney *U*-test or unpaired *t*-test was used to compare two independent groups. Before performing a unpaired *t*-test, normality of distribution for each group was tested by Shapiro–Wilk test. An ANOVA, Bonferroni correction or FDR was performed when more than two groups were compared. The variances between groups were assumed to be similar. Throughout the study, *P*<0.05 was considered statistically significant, other than the definitions of visually responsive and selective cells (see Data analysis section). The sample size *n* was defined either as the number of animals or images. No statistical methods were used to pre-determine sample sizes, but our sample sizes are similar to those generally employed in the field.

### Data availability

The data that support the findings of this study are available from the corresponding author upon request.

## Additional information

**How to cite this article:** Kondo, S. *et al*. Mixed functional microarchitectures for orientation selectivity in the mouse primary visual cortex. *Nat. Commun.* **7,** 13210 doi: 10.1038/ncomms13210 (2016).

## Supplementary Material

Supplementary Information Supplementary Figures 1 - 10, Supplementary Tables 1 - 4 and Supplementary References
