# Supplementary material for: Mixed functional microarchitectures for orientation selectivity in the mouse primary visual cortex
Source: Nat Commun. 2016 Oct 21;7:13210. doi: 10.1038/ncomms13210 (PMC5078743; doi:10.1038/ncomms13210)
Supplement: Supplementary Information — Supplementary Figures 1 - 10, Supplementary Tables 1 - 4 and Supplementary References [file ncomms13210-s1.pdf]

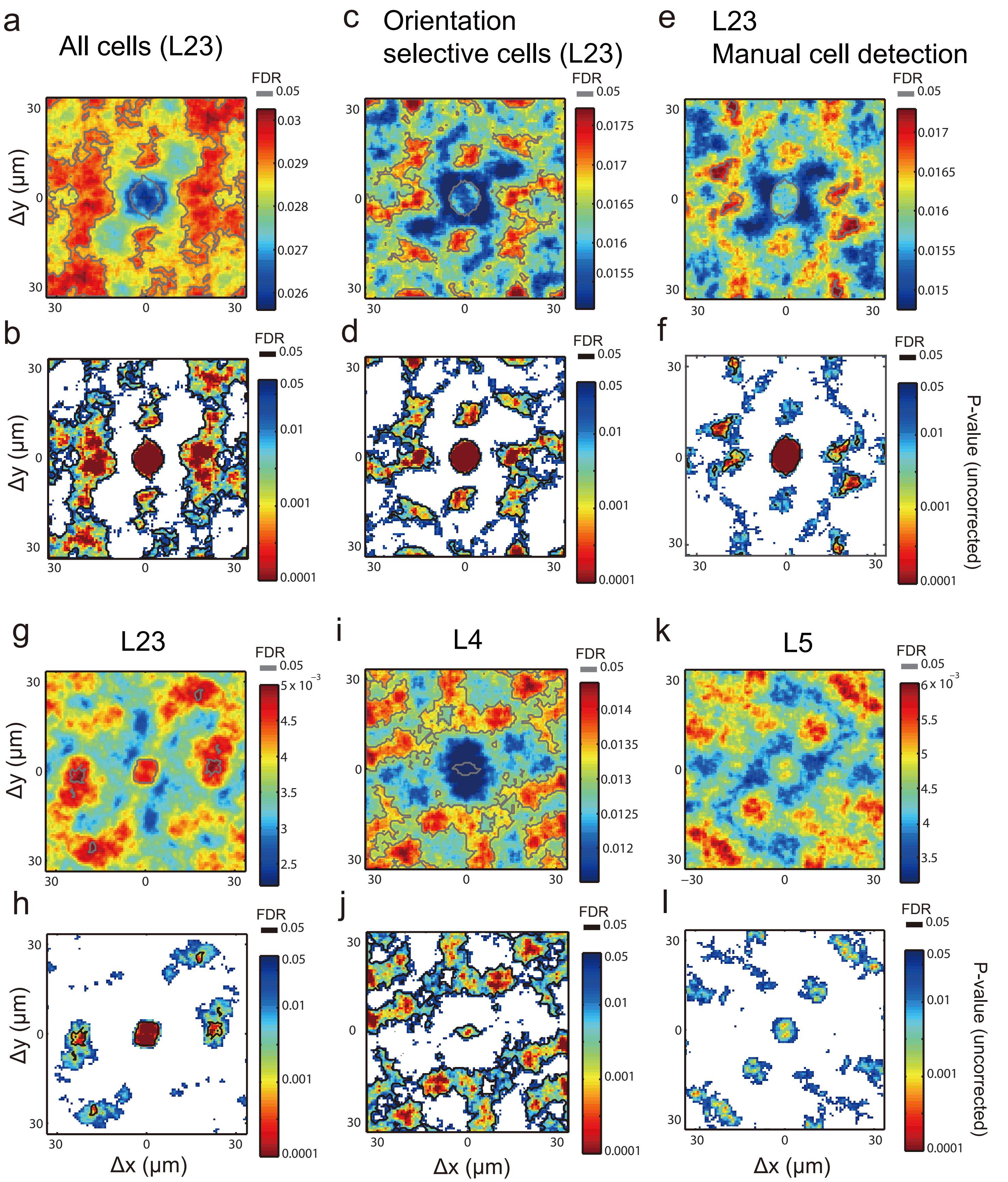

Kondo, Yoshida and Ohki, Supplementary Figure 1

Supplementary Figure 1. Regularly spaced vertical structures in layers 2/3, 4, and 5

a,b The 2-D probability density map (**a**) and corresponding p-value map (**b**) (see Methods) obtained from all the neighboring cells shows several blurry hot spots. In this case, peak and valley probabilities were about 0.029 and 0.027, respectively (corresponding to 2.3 and 2.1 cells within a range of 5 μm radius). **c,d** Probability density map (**c**) and corresponding p-value map (**d**) obtained from orientation selective cells (the same as in Fig1e). In this case, the probability density of orientation selective cells around these hotspots and neighboring valleys are about 0.017 and 0.015 cells /μm^2, corresponding to 1.34 cells and 1.18 cells within a range of 5 μm radius. Hotspots of probability density map from all the cells were wider than those obtained from the orientation selective cells (**a-d**). We hypothesized that this blurriness may result from GABAergic cells that do not belong to minicolumns. It has been suggested that minicolumns are derived from excitatory neurons that migrate through the radial glial fibers[1]. It is also reported that developmental origin and migration pattern of GABAergic cells are different from excitatory cells[2]. Thus, GABAergic neurons may not locate along the vertical alignment of excitatory neurons. Because it was reported that GABAergic neurons are broadly tuned for orientation[3,4], neurons that pass our thresholds for orientation-selective cells are most likely excitatory neurons. **e,f** To confirm that the regularity does not depend on the cell detection method, we detected the cells manually and calculated the density map (**e**) and corresponding p-value map (**f**). Although the density maps were not identical between semi-automatic and manual detection data, the spatial patterns were similar (correlation coefficient = 0.7). Therefore, the regularity in cell position was maintained in the manually detected data. **g-l** Representative maps that only orientation selective cells were used from layer 2/3 (**g**), layer 4 (**i**), and from layer 5 (**k**), and corresponding p-value maps (**h,j,l**). All examples show spatially localized hotspots around the origin. This tendency was consistent in many animals, although statistical significances were not detected in some cases (see **k** and **l**, FDR adjusted $p > 0.05$). Spatial pattern of hotspot was variable across animals (**c-l**). Gray contours in (**a,c,e,g,i**) indicate statistically significant area (FDR-adjusted $p < 0.05$). Black contours in (**b,d,f,h,j**) indicate significant area (FDR-adjusted $p < 0.05$).

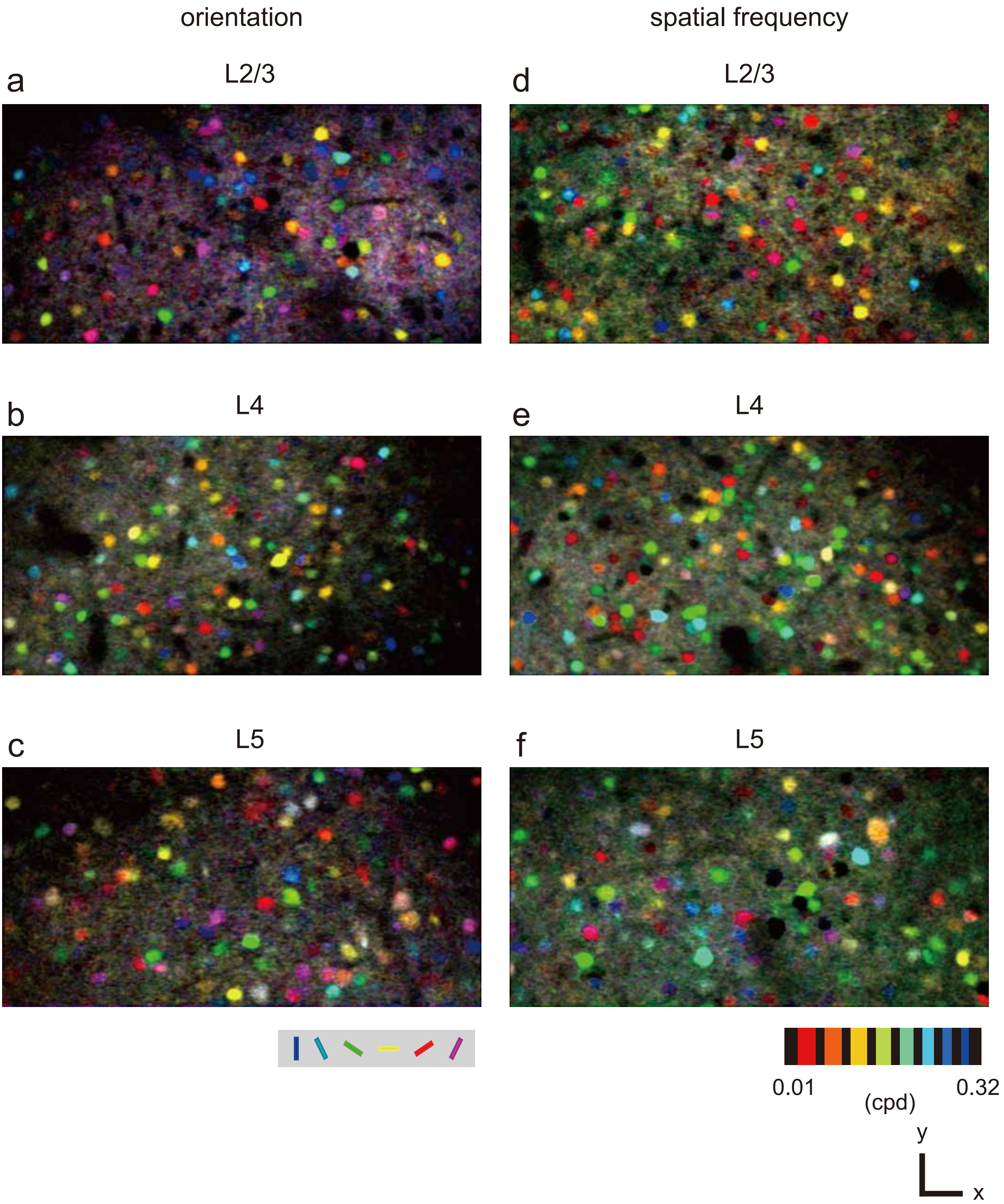

Kondo, Yoshida and Ohki, Supplementary Figure 2

Supplementary Figure 2. Salt and pepper organization is also observed in deeper layers
Pixel-based color maps of an x–y plane image are shown for orientation (**a**-**c**) and for SPF (**d**-**f**). As has been demonstrated in our previous study, neurons with similar response selectivity are scattered in a salt and pepper manner in the upper cortical layer (**a**: orientation; **d**: SPF). The present study showed that this salt and pepper organization is also observed in deeper layers (layers 4 and 5) for both visual functions (**b**, **c**: orientation; **e**, **f**: SPF). Scale bars 30 μm.

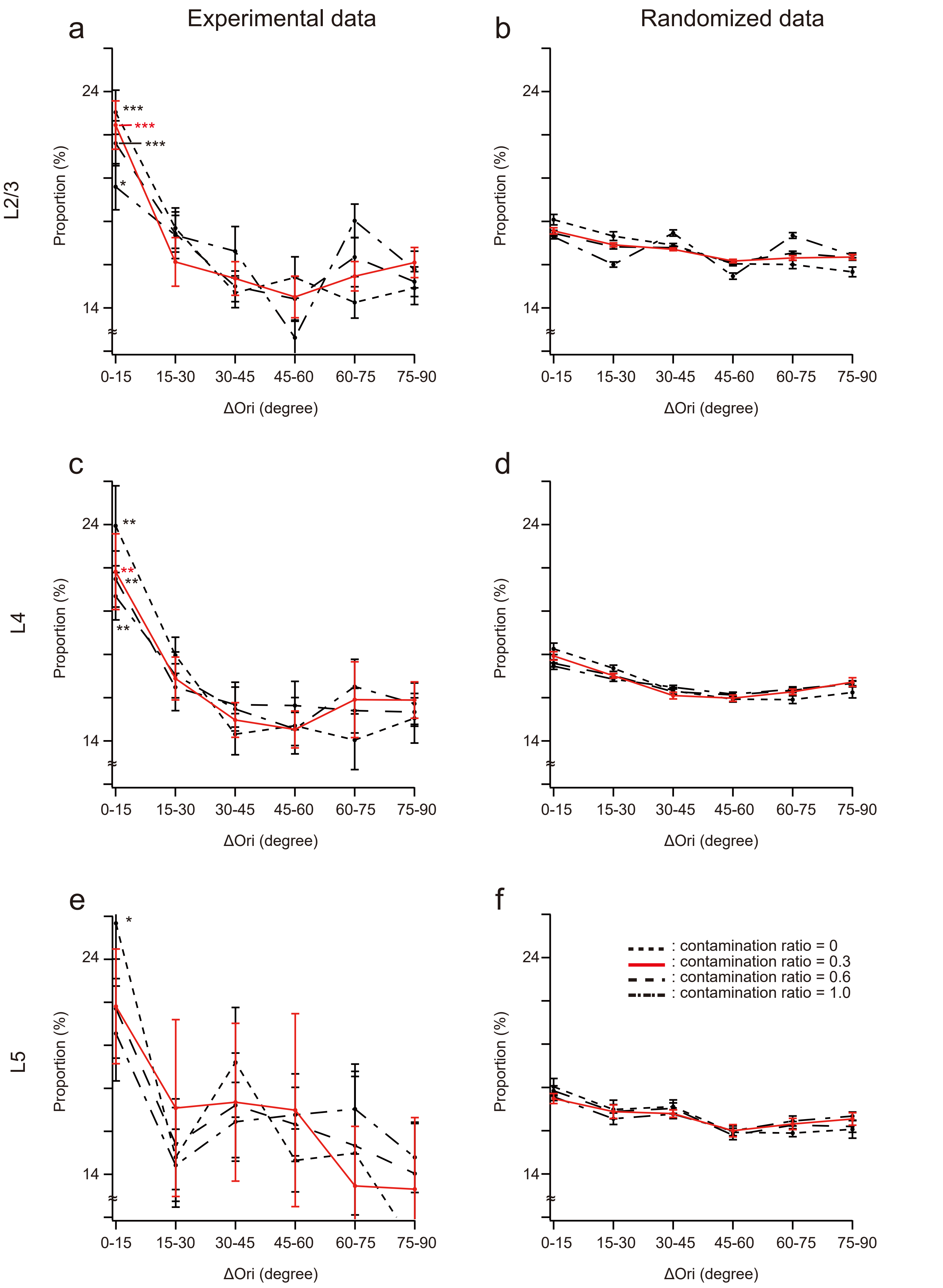

Kondo, Yoshida and Ohki, Supplementary Figure 3

Supplementary Figure 3. Neuropil contamination does not affect on the measurement of functional clustering of minicolumns

To address whether the functional clustering of mini-columns is an artifact of neuropil contamination by dendritic bundles (micro-columns) that pass closely to the layers 2/3 or 4 cells, we have changed neuropil contamination ratio from zero to one (100%), and repeated the analysis of the functional clustering of mini-columns, similar to Figure 3a. **a,c,e** The proportion of neurons with similar orientation selectivity within a minicolumn (radius = 5 μm) along the 100 μm length. The difference in orientation selectivity was calculated between cell pairs and divided into equal-interval bins (15°) at different neuropil contamination ratio (0, 0.3 0.6, and 1.0). The proportion of cell pairs was plotted as a function of bins. **b,d,f** We repeated the same analysis for the randomized maps (1,000 times) and plotted the proportion of Δori bins. Even when we assume 100% of contamination of neuropil signal, functional clustering is statistically significant compared with randomized distribution. Significant differences between experimental and randomized map were observed in the Δori = 0–15° bin in layer 2/3 ($p < 0.001$: neuropil contamination ratio = 0, 0.3, and 0.6, $p < 0.05$: neuropil contamination ratio = 1.0, n=26 images from 14 mice), in layer 4 ($p < 0.01$, n=14 images from 10 mice) and in layer 5 ($p < 0.05$ at neuropil contamination ratio = 0, n=10 images from 6 mice). Red: neuropil contamination ratio = 0.3 (the same value as in Figure 3); black: neuropil contamination ratio =0 (short dotted line), 0.6 (long dotted line), 1.0 (combination of short and long dotted lines).

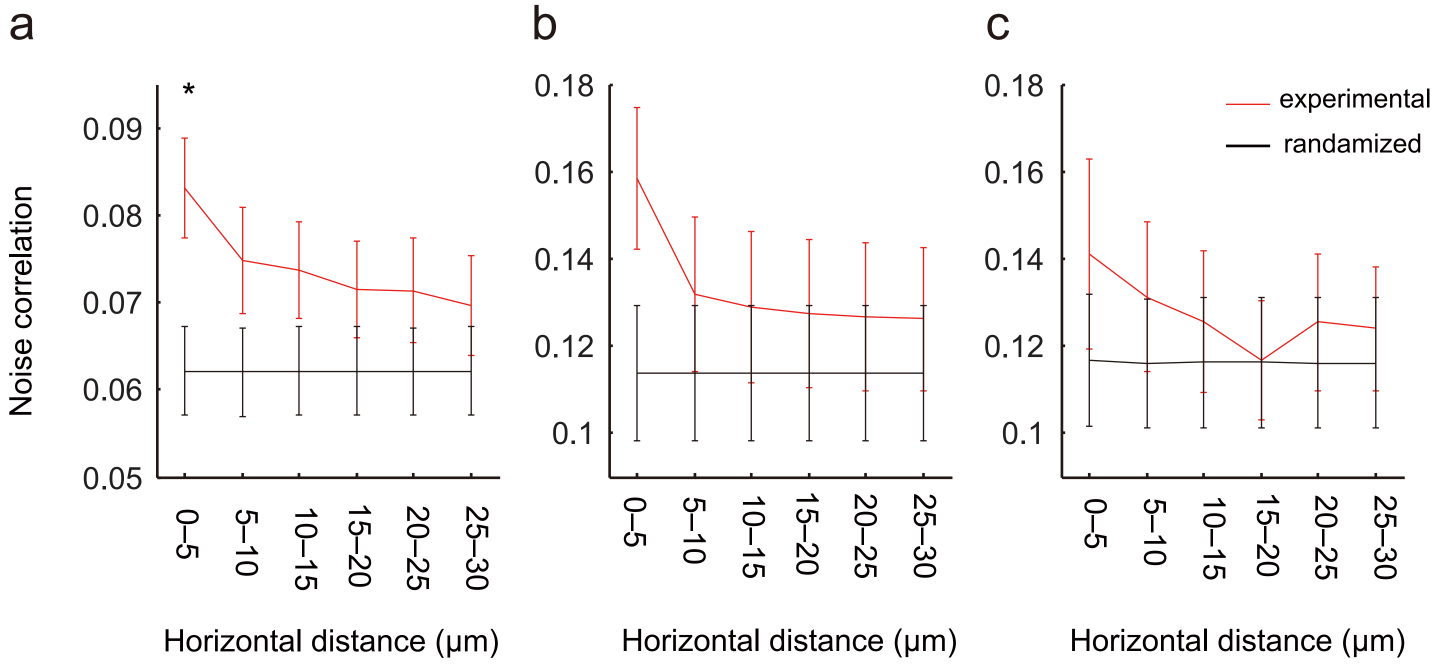

Kondo, Yoshida and Ohki, Supplementary Figure 4

Supplementary Figure 4. Noise-correlation analysis suggest the functional connectivity of cells within a minicolumn.

To test whether cells belonging to the same minicolumn tend to work together, we investigated the noise-correlation of cells within a minicolumn. Noise-correlations of neighboring cells were calculated as a function of horizontal distances between the cells. We repeated the same analysis for the randomized data. We observed the similar tendency as we observed in ΔOri analysis (Fig. 3b). The noise-correlation rapidly decreased in cell pairs at 5-10 μm in the horizontal direction in all the layers (a: layer 2/3, b: layer 4, c: layer 5). These results suggest that functional connectivity between cells could be higher in minicolumn. * $p < 0.05$. red: experimental data; black: randomized data.

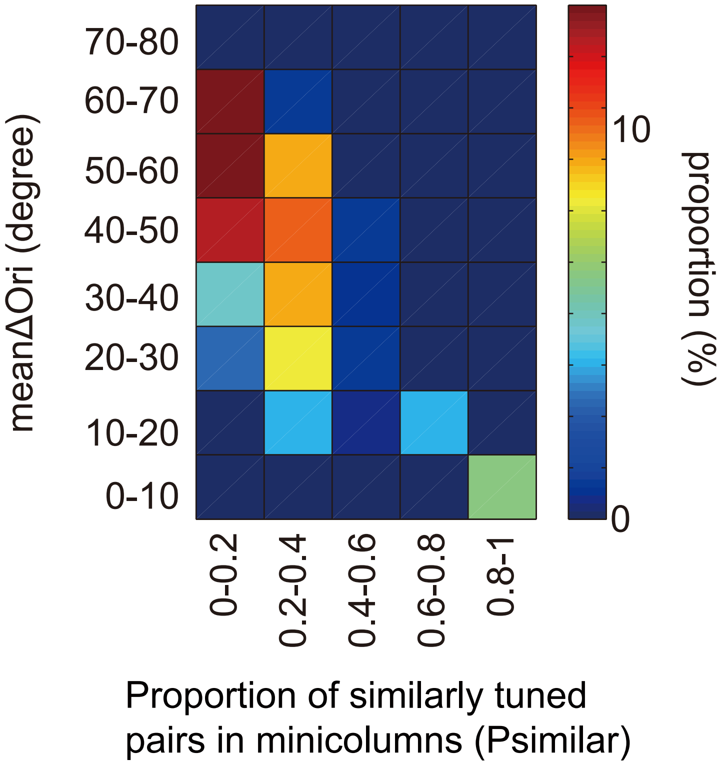

Kondo. Yoshida and Ohki, Supplementary Figure 5

Supplementary Figure 5. Relation between Psimilar and meanΔori

The relation between Psimilar and meanΔOri is shown as a matrix histogram. The proportion of minicolumns in each bin was color-coded. Overall, Psimilar and meanΔori were negatively correlated. When all the neurons in the minicolumns were similarly tuned, the correspondence between high Psimilar (0.8-1.0) and low meanΔori (0-10 degrees) was precise. However, when some neurons in the minicolumns were differently tuned (Psimilar: 0-0.8, meanΔori: 10-70 degrees), the correspondence between two measures became loose. Therefore, both measures may complementary describe the similarity of preferred orientations in minicolumns, and Psimilar corresponds more directly to the analyses in Figure 3.

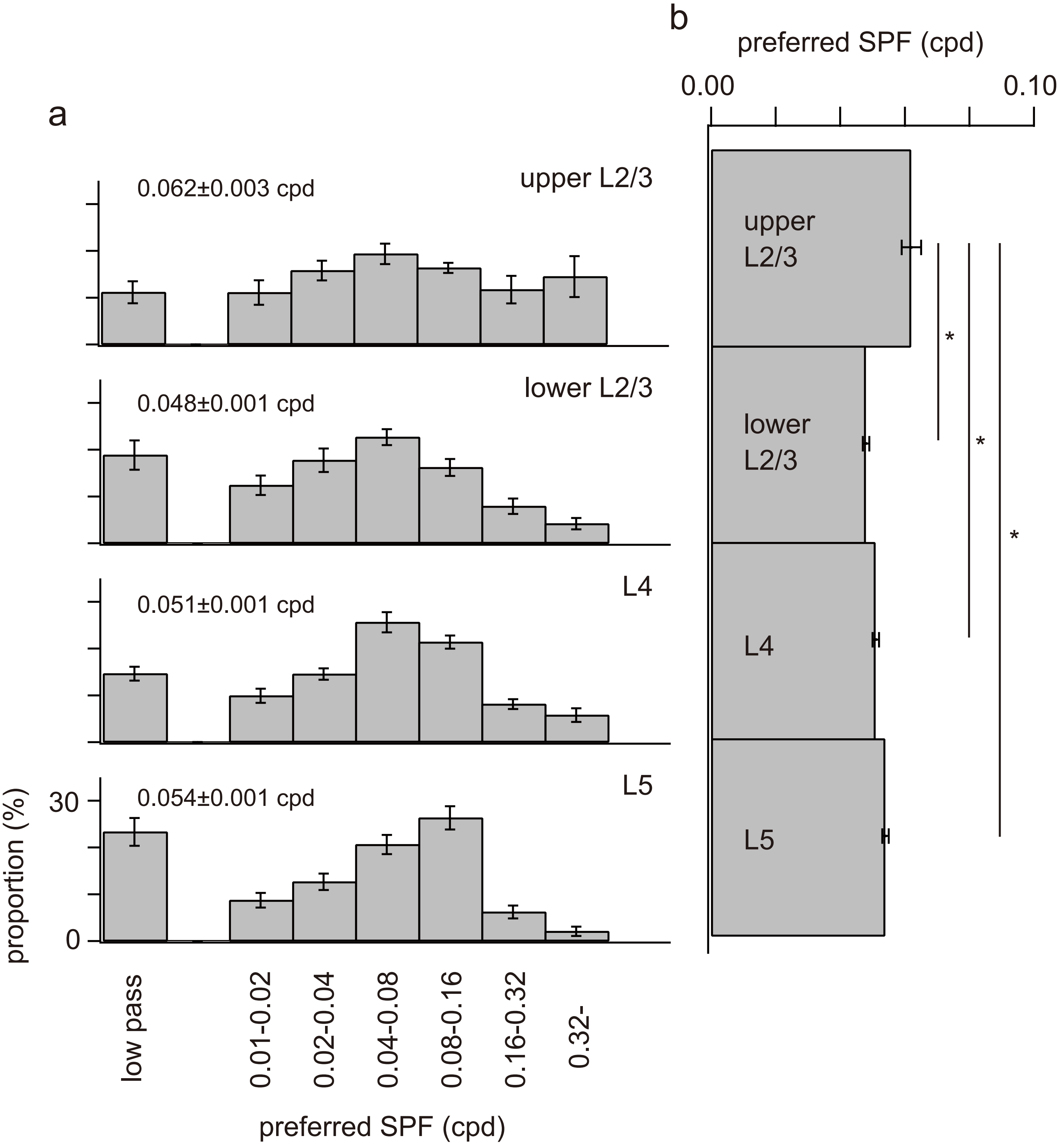

Kondo, Yoshida and Ohki, Supplementary Figure 6

Supplementary Figure 6. Similar SPF tuning property across different layers

a Distribution of the preferred SPF tuning property is shown for different layers (upper and lower layer 2/3, layer 4, and layer 5). We observed a low-pass- and a band-pass-filtered type. The distribution of the preferred SPFs of neurons in a local volume were not uniform, but biased toward a particular SPF. **b** Averaged preferred SPF tuning property for different layers. The differences between upper layer 2/3 and layers lower 2/3, 4 and 5 are statistically significant ($p < 0.05$, n=15 images from 10 mice for layer 2/3, n=12 images from 9 mice for layer 4, n=8 images from 5 mice for layer 5, Mann–Whitney U-test with Bonferroni correction) but between other layers are not statistically significant ($p > 0.05$, n=15 images from 10 mice for layer 2/3, n=12 images from 9 mice for layer 4, n=8 images from 5 mice for layer 5, Mann–Whitney U-test with Bonferroni correction). Thus, the overall mean preferred SPF was similar among different layers. Because we did not distinguish pyramidal neurons and interneurons, the analyzed neurons may contain both types[3].

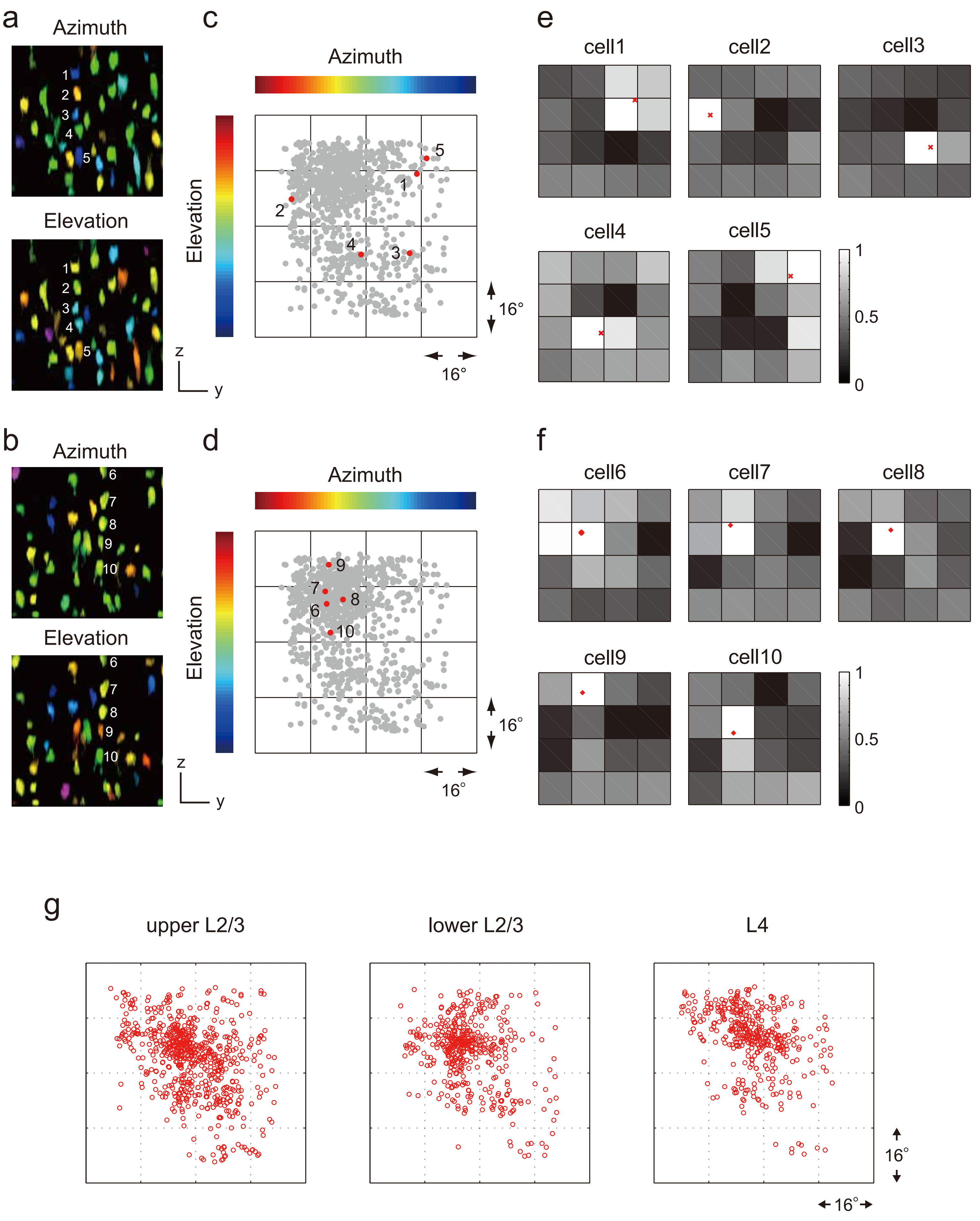

Kondo, Yoshida and Ohki, Supplementary Figure 7

Supplementary Figure 7. Vertical alignment of cells with similar receptive field position selectivity

The retinotopic organization of the visual cortex of mice has been demonstrated as a macroscopic structure by extracellular electrical recording[5,6] and by intrinsic optical imaging[7,8]. By contrast, the microarchitecture revealed by *in vivo* two-photon calcium imaging showed a scattered arrangement of retinotopic positions[9-11]. We examined whether there is any clustering of retinotopic positions specific to minicolumns. **a,b** We reconstructed color-coded three-dimensional maps of the azimuth and elevation of the preferred retinotopic positions. The azimuth (upper panel) and elevation (lower panel) maps were resliced in the x–z planes. Scale bars 30 μm. **c,d** The retinotopic positions of all the cells (gray circles) and individual cells indicated in **a** or in **b** (red circles with a number) are plotted. The retinotopic positions were widely distributed in some minicolumns (**a** and **c**, cells 1–5), whereas these positions were more similar in other minicolumns (**b** and **d**, cells 6–10). **e,f** Normalized calcium signal changes are shown with a gray scale for each 4 × 4 grid stimulation. The peak position of surface fitting with a two-dimensional Gaussian function is indicated as a red closed circle (preferred retinotopic position). **g** Retinotopic positions of all the cells within a volume are plotted as red circles within 4 × 4 grids. The results from consecutive images along the z-axis from upper and lower layer 2/3 to layer 4 are shown. The numbers of cells plotted (above thresholds) were 664 (upper layer 2/3), 445 (lower layer 2/3), and 397 (layer 4).

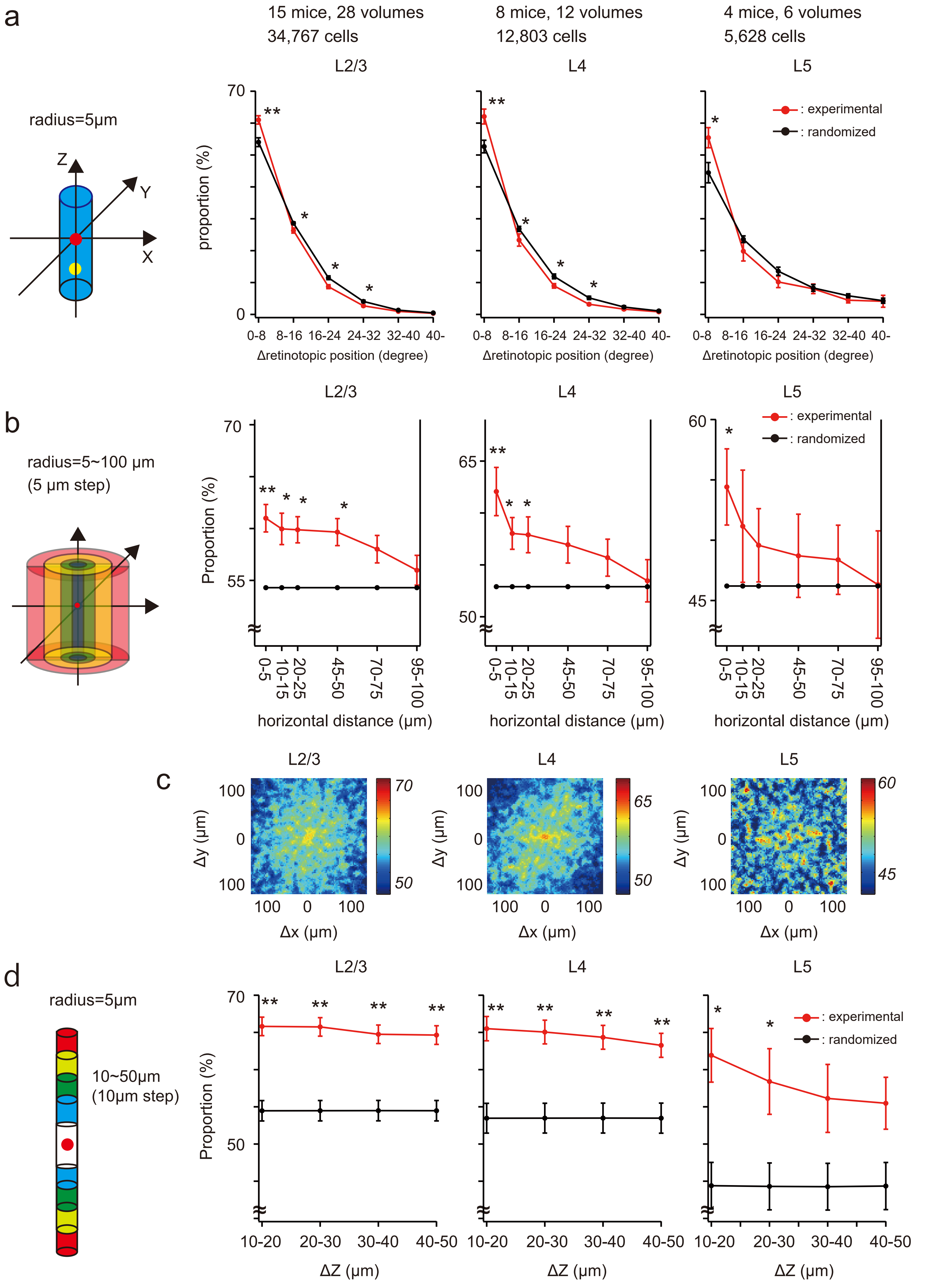

Kondo, Yoshida and Ohki, Supplementary Figure 8

Supplementary Figure 8. Proportion of cells with a similar retinotopic position within a minicolumn

a We quantitatively evaluated the similarity between the retinotopic positions of neurons in the same minicolumn. The proportion of cells with similar retinotopic positions was calculated within a minicolumn of 5 μm radius for layers 2/3, 4, and 5. The difference in retinotopic position selectivity was calculated between cell pairs and divided into equal-interval bins (8°). The proportion of cell pairs was plotted as a function of bins. As expected from the existence of the macroscopic retinotopic position maps[7,8] and from our observation that the retinotopic positions of neurons in a local volume tended to concentrate in a specific position, a high proportion of neurons with similar retinotopic positions was observed within the same minicolumn in layers 2/3, 4, and 5. **b** We investigated whether this significant difference is restricted to the same minicolumn by examining the proportion of cell pairs with similar retinotopic positions (within 8° apart) as a function of the horizontal distance between the pairs, as we did for orientation and for SPF selectivity (Fig. 3b and Fig. 5d). The proportion showed moderately high values at 0–5 μm (0.12 for layer 2/3 and approximately 0.17 for layers 4 and 5) and slowly decreased as a function of the distance between cell pairs, suggesting that the similarity in retinotopic positions is not specific to minicolumns but is primarily due to clustering at a larger spatial scale, most likely corresponding to the global retinotopic map. **c** A proportion map of cell pairs with similar preferred retinotopic positions was plotted as a function of relative horizontal positions between cell pairs. The map shows the horizontal spread of cell pairs with similar retinotopic positions in layers 2/3 and 4, which is less evident in the noisy map of layer 5. The color bar shows the proportion (%) on the left and the SI on the right. **d** Vertical distribution of similarly tuned cells. We chose cell pairs within 5 μm in the horizontal direction and at 10–20, 20–30, 30–40, or at 40–50 μm in the vertical direction and calculated the proportion of similarly tuned cells (Δretinotopic position <8°). We observed almost no difference across vertical distances, suggesting that neurons with similar retinotopic positions are distributed evenly along the depth axis.

These analyses suggest that retinotopic position maps are coarsely clustered. The scale of clustering is much larger than that of minicolumns and likely corresponds to a global retinotopic map. ** $p < 0.01$ and * $p < 0.05$. Red: experimental data; black: randomized data.

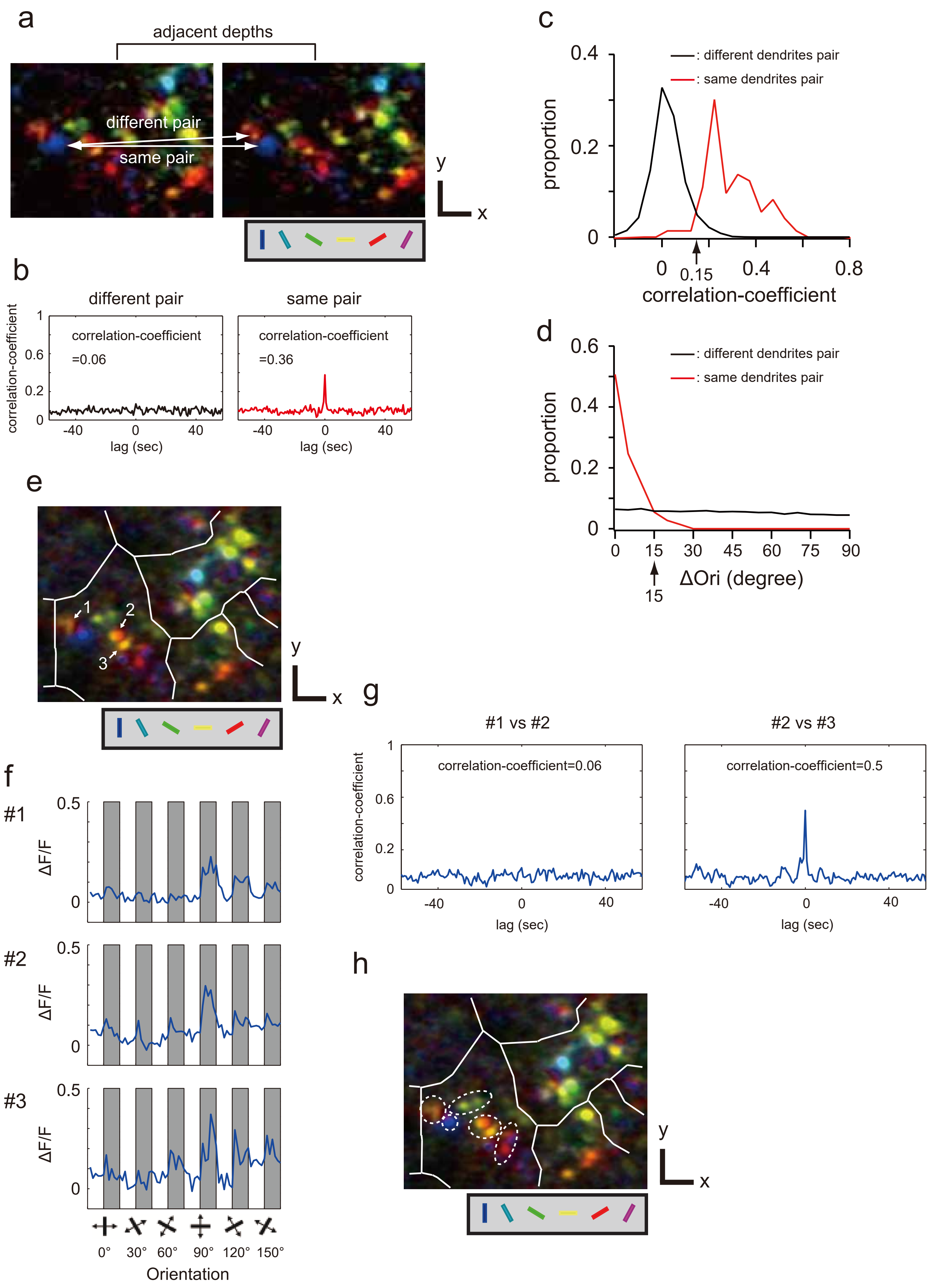

Kondo, Yoshida and Ohki, Supplementary Figure 9

Supplementary Figure 9. Elimination of dendrites within a bundle that potentially originated from the same neuron

Because apical dendrites of layer 5 neurons occasionally form branches (although the branching is relatively less in the primary visual cortex of rodents[12,13]), some dendrites in the same bundle may belong to the same cell. We could not anatomically identify the branched dendrites from the same soma because dendrites loaded with OGB-1 AM were not bright enough to follow thin dendrites to branching points. We physiologically identified dendrites belonging to the same cell by finding highly correlated calcium signals and similar visual selectivity in pairs of dendrites (see Methods for details) and eliminated the duplicated counts. **a** We chose two adjacent depth images from three-dimensional data and identified the same dendrites in the two images. Scale bars 10 μm. **b** We generated shuffle-corrected time courses from each dendrite and calculated cross-correlograms and correlation coefficients between the same and different dendrite pairs. Cross-correlograms show a sharp peak at time zero between the same dendrite pair, whereas the peak is almost absent between different dendrite pairs. A histogram of correlation coefficients shows a clear separation between the same and different dendrites. **c** Differences in the orientation selectivity (Δori) between the same and different dendrites were calculated, and the proportion was plotted. **d** We set the threshold value to distinguish between the dendrite pairs of the same or different cells at 0.15 and 15° for the correlation coefficient and for Δori, respectively, as shown in **c** and in **d**. We eliminated dendrites within a bundle that potentially originated from the same neuron. An example is shown from the same data as that in Fig. 6. Three dendrites with similar orientation preferences were selected (**e**), and their averaged time courses are shown in **f**. **g** Shuffle-corrected time courses were generated, and cross-correlograms were calculated. A strong peak at time zero was observed in one pair (**g**, right) but not in the other (**g**, left), and the correlation coefficients were 0.5 and 0.06, respectively. In this case, one dendrite was eliminated from a pair (#2 and #3) that showed a correlation coefficient greater than 0.15. After we repeated this analysis, the dendrites from different neurons were encircled with white dots (**h**). Scale bars 10 μm in **e** and **h**.

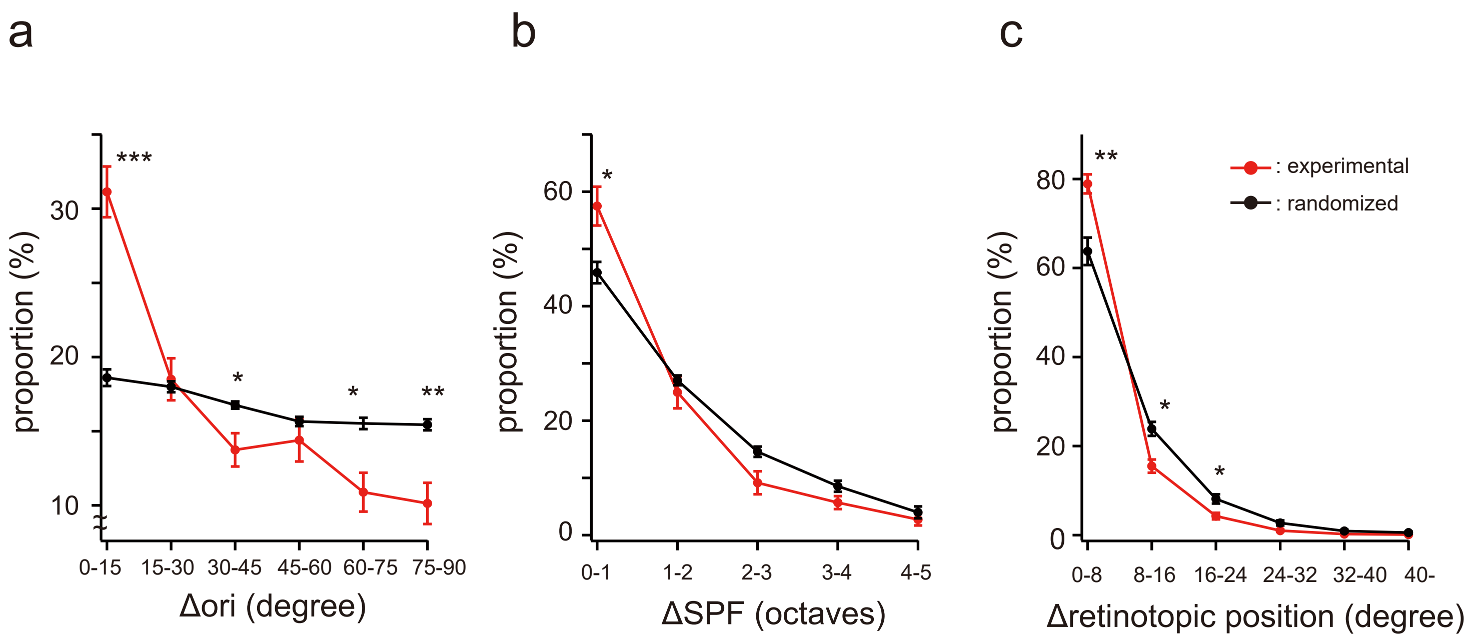

Kondo, Yoshida and Ohki, Supplementary Figure 10

Supplementary Figure 10. Proportion of dendrites with similar response properties within a bundle

The plots before eliminating the duplicated counts of dendrites are shown. The proportion of dendrites with similar orientation (**a**), SPF (**b**), and retinotopic position (**c**) selectivity within a bundle before the correction. The proportion of dendrites with similar response selectivity is higher in all the visual functions compared with the results after the correction; however, the tendency is not significantly altered (see Fig. 7a-c).

Supplementary Table 1
Summary of the responses to orientation stimulations

layer	N. mice	N. volumes	N. cells	Responsive (%)	Selective (%)
L2/3	14	26	31,890	48.9	33.5
L4	10	14	15,282	57.1	38.6
L5	6	10	9,566	28.1	19.4

The criteria for responsive and selective cells are described in Methods.

Supplementary Table 2
Summary of the responses to spatial frequency stimulations

layer	N. mice	N. volumes	N. cells	Responsive (%)	Selective (%)
L2/3	10	15	19,645	47.2	33.7
L4	9	12	13,767	51.3	38.4
L5	5	8	7,549	28.1	21.3

The criteria for responsive and selective cells are described in Methods.

Supplementary Table 3
Summary of the responses to retinotopic position stimulations

layer	N. mice	N. volumes	N. cells	Responsive (%)	Selective (%)
L2/3	15	28	34,767	50.3	48.3
L4	8	12	12,803	58.5	55.7
L5	4	6	5,628	32.6	30.6

The criteria for responsive and selective cells are described in Methods.

Supplementary Table 4.

Summary of the responses of dendrites to orientation, spatial frequency, and retinotopic position stimulations

Visual functions	N. mice	N. images	N. dendrites	Responsive (%)	Selective (%)
Orientation	16	42	10,544	38.1	23.0
SPF	13	25	6,521	38.6	20.7
Retinotopic positions	11	26	7,746	44.1	36.2

The criteria for responsive and selective cells are described in Methods.

SUPPLEMENTARY REFERENCES

1. Rakic, P. Guidance of neurons migrating to the fetal monkey neocortex. *Brain Res.* **33**, 471–476 (1971).

2. Anderson, S.A., Eisenstat, D.D., Shi, L. & Rubenstein, J.L. Interneuron migration from basal forebrain to neocortex: dependence on Dlx genes. *Science* **278**, 474-476 (1997).

3. Kerlin, A.M., Andermann, M.L., Berezovskii, V.K., & Reid, R.C. Broadly tuned response properties of diverse inhibitory neuron subtypes in mouse visual cortex. *Neuron* **67**, 858-71 (2010).

4. Sohya, K., Kameyama, K., Yanagawa, Y., Obata, K., & Tsumoto, T. GABAergic neurons are less selective to stimulus orientation than excitatory neurons in layer II/III of visual cortex, as revealed by in vivo functional Ca2+ imaging in transgenic mice. *J. Neurosci.* **27**, 2145-9 (2007).

5. Drager, U.C.. Receptive fields of single cells and topography in mouse visual cortex. J. Comp. Neurol. *160,* 269-90, (1975).

6. Wagor, E., Mangini, N.J., & Pearlman, A.L. Retinotopic organization of striate and extrastriate visual cortex in the mouse. *J. Comp. Neurol.* **193**, 187-202 (1980).

7. Schuett, S., Bonhoeffer, T., & Hubener, M. Mapping retinotopic structure in mouse visual cortex with optical imaging. *J. Neurosci.* **22**, 6549-59, (2002).

8. Kalatsky, V.A. & Stryker, M.P. New paradigm for optical imaging: temporally encoded maps of intrinsic signal. *Neuron* **38**, 529-45, (2003).

9. Bonin, V., Histed, M.H., Yurgenson, S., and Reid, R.C.. Local diversity and fine-scale organization of receptive fields in mouse visual cortex. J. Neurosci. *31,* 18506-21, (2011).

10. Mrsic-Flogel, T.D., Hofer, S.B., Creutzfeld, C., Cloez-Tayanari, I., Changeux, J.P., Bonhoeffer, T., & Hubener, M. Altered map of visual space in the superior colliculus of mice lacking early retinal waves. *J. Neurosci.* **29**, 6921-8 (2005).

11. Smith, S.L., & Hausser, M. Parallel processing of visual space by neighboring neurons in mouse visual cortex. *Nat. Neurosci.* 13, 1144-9 (2010).

12. Kasper, E.M., Larkman, A.U., Lubke, J., and Blakemore, C.. Pyramidal neurons in layer 5 of the rat visual cortex. I. Correlation among cell morphology, intrinsic electrophysiological properties, and axon targets. J. Comp. Neurol. *339,* 459-74, (1994).

13. Tsiola, A., Hamzei-Sichani, F., Peterlin, Z., and Yuste, R.. Quantitative morphologic classification of layer 5 neurons from mouse primary visual cortex. J. Comp. Neurol. *461,* 415-28, (2003).
